# A multivalent RNA affinity tag enables selective purification of tagged RNAs and bound proteins

**DOI:** 10.1093/nar/gkag735

**Published:** 2026-07-30

**Authors:** Ki Sung Park, Sourav Kumar Dey, Rohit Nalavade, Aashiq H Mirza, Qian Hou, Mateo A Martinez Roque, Maxim Oleynikov, Jacob L Litke, Samie R Jaffrey

**Affiliations:** Department of Pharmacology, Weill Cornell Medicine, Cornell University, New York, NY 10065, United States; Department of Pharmacology, Weill Cornell Medicine, Cornell University, New York, NY 10065, United States; Department of Pharmacology, Weill Cornell Medicine, Cornell University, New York, NY 10065, United States; Department of Pharmacology, Weill Cornell Medicine, Cornell University, New York, NY 10065, United States; Department of Pharmacology, Weill Cornell Medicine, Cornell University, New York, NY 10065, United States; Department of Pharmacology, Weill Cornell Medicine, Cornell University, New York, NY 10065, United States; Department of Pharmacology, Weill Cornell Medicine, Cornell University, New York, NY 10065, United States; Department of Pharmacology, Weill Cornell Medicine, Cornell University, New York, NY 10065, United States; Department of Pharmacology, Weill Cornell Medicine, Cornell University, New York, NY 10065, United States

## Abstract

Efficient purification of specific RNAs from lysates remains a major challenge. Existing methods, such as MS2 tagging or oligonucleotide hybridization, require protein immobilization or sequence-specific hybridization, limiting scalability and compatibility with diverse RNAs. Current approaches often have low recovery, exhibit slow kinetics, and generate background contamination, limiting their use in RNA–protein interaction studies. To overcome these limitations, we developed FS2, an RNA sequence that binds Sephadex beads with high affinity, thus acting as an affinity tag for protein-free, rapid, and simple RNA purification. FS2 consists of two copies of the dextran-binding D8 aptamer embedded within the highly stable F30 three-way junction RNA scaffold, which promotes aptamer folding and enhances avidity. Systematic optimization revealed that FS2 exhibits rapid binding kinetics, efficient purification, and efficient elution under mild conditions (50°C, 10 mM EDTA), representing substantial improvements over existing RNA purification systems. We validated the utility of FS2 by purifying an FS2-tagged *MYC* mRNA fragment from HEK293T cells and demonstrating that *N*^6^-methyladenosine (m^6^A)-containing mRNAs can be pulled down along with the m^6^A-binding protein YTHDF2. Overall, FS2 provides a highly simple and efficient RNA-based affinity tag for recovering RNA from complex mixtures and lysates for diverse applications.

## Graphical Abstract

**Graphical Abstract:**
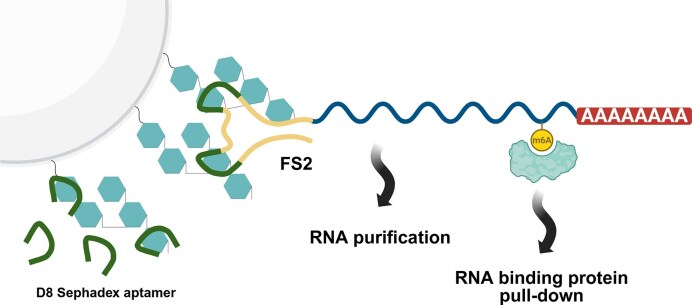

## Introduction

In many cases, it is useful to be able to rapidly and efficiently pull down specific RNA sequences for purifying the RNA itself or its bound proteins. A major RNA pulldown method relies on hybridization-based capture using complementary oligonucleotides [1–3]. However, this method can be complex since it requires custom-designed oligonucleotides with optimized wash conditions that are carefully designed to match the T_m_ of the oligonucleotides. Additionally, the hybridization and wash conditions are often not compatible with retaining native RNA structures and protein interactions [4]. Aptamer-based methods have also been developed. These involve incorporating MS2 hairpins followed by purification on beads containing MS2 coat protein (MCP) [5, 6]. A similar method involves the streptavidin aptamer [7], which binds immobilized streptavidin. However, these require the immobilization of recombinant proteins onto affinity matrices, and have constraints such as limited binding capacity, increased background binding, and the need for protein purification steps [6]. Although the protein-binding aptamer tags are useful, these challenges underscore the need for RNA-based affinity tags that can be incorporated into any sequence and can allow highly efficient purification at low cost.

An alternative approach for RNA pulldown utilizes RNA aptamers that directly interact with small-molecule matrices. One such aptamer that was developed for RNA purification was the Sephadex-binding D8 aptamer, which binds to Sephadex beads [8]. Sephadex beads are composed of a specific type of dextran that contains α-1,6-linked glucose residues. Unlike protein-based affinity tags, the D8 aptamer does not require an immobilized binding partner, since the aptamer binds to the matrix itself. This bypasses the need for making an affinity matrix, and conveniently provides an exceptionally large number of binding sites on the Sephadex matrix. Although the D8 aptamer has been used to purify ribonucleoprotein complexes—such as yeast RNase P [9], it is not widely used for RNA purification. This suggests that this aptamer may have limitations that limit its broader utilization. Conceivably, enhancing the RNA-purification ability of the D8 aptamer could lead to a reliable and broadly applicable system for RNA pulldown.

In this study, we explored the D8 aptamer and found that its binding function was markedly impaired when it was inserted into various RNAs. To overcome this, we developed a new affinity tag in which the D8 aptamer was inserted into an F30 RNA-folding scaffold, enabling D8 folding and function in diverse sequence contexts. This system was further improved by inserting two D8 aptamers into F30, resulting in a bivalent aptamer to increase avidity. The resulting RNA, termed FS2, led to improved effective affinity and RNA recovery from lysates, without compromising selective elution of tagged RNAs. We show that FS2-tagged RNAs can be purified from mixtures and cellular lysates, and can be used for pulldown of associated RNA-binding proteins.

## Material and methods

### Plasmid construction and RNA synthesis

FS2-tagged RNA constructs were generated by cloning the FS2 sequence into the appropriate position of each target RNA construct. Depending on the construct design, FS2 was placed at the 5′ end, 3′ end, or within circular RNA constructs. The promoter, tag position, and nucleotide sequence information for each construct are provided in Supplementary Table S1. For *in vitro* transcription, template DNA was amplified by PCR using primers containing the T7 promoter sequence. The resulting PCR product was transcribed using the AmpliScribe™ T7 High Yield Transcription Kit (LGC Biosearch Technologies, Petaluma, CA, USA) according to the manufacturer’s instructions. Transcribed RNA was then purified using the RNA Clean & Concentrator™-25 Kit (Zymo Research, Irvine, CA, USA).

For the generation of circular RNA constructs, FS2-tagged linear RNA was processed using the Tornado system, as described in our previous study [10].

### Cell culture and transfection

HEK293T cells were maintained in high-glucose Dulbecco’s Modified Eagle Medium (DMEM; Gibco, Thermo Fisher Scientific, Waltham, MA, USA) supplemented with 10% (v/v) fetal bovine serum (FBS; Gibco, Thermo Fisher Scientific, Waltham, MA, USA) and 1% (v/v) penicillin-streptomycin (Thermo Fisher Scientific, Waltham, MA, USA). Cells were cultured at 37°C in a humidified incubator with 5% CO₂.

Mouse embryonic stem cells (mESCs) were maintained in KnockOut™ DMEM (Gibco, Thermo Fisher Scientific, Waltham, MA, USA), supplemented with 15% (v/v) FBS (Gibco, Thermo Fisher Scientific, Waltham, MA, USA), 1% (v/v) penicillin-streptomycin (Thermo Fisher Scientific, Waltham, MA, USA), 2 mM L-glutamine (Thermo Fisher Scientific, Waltham, MA, USA), 55 μM β-mercaptoethanol (Gibco, Thermo Fisher Scientific, Waltham, MA, USA), 1 × MEM non-essential amino acids (NEAA) (Gibco, Thermo Fisher Scientific, Waltham, MA, USA), and 1 000 U/ml Murine leukemia inhibitory factor (LIF) (ESGRO, Millipore, Burlington, MA, USA). For maintaining pluripotency, cells were cultured with 3 μM CHIR99021 (SML1046, Sigma-Aldrich, St. Louis, MO, USA) and 1 μM PD0325901 (A3013, APExBIO, Houston, TX, USA).

Cells were cultured on gelatin-coated tissue culture plates in a humidified incubator at 37°C with 5% CO₂. Gelatin-coated plates were prepared by incubating tissue culture dishes with EmbryoMax® 0.1% Gelatin Solution (Millipore Sigma, Burlington, MA, USA) at 37°C for 30 min, followed by removal of excess gelatin.

For transfection, cells were seeded 1 day before and transfected with FS2-tagged constructs using FuGENE® HD Transfection Reagent (Promega, Madison, WI, USA) according to the manufacturer’s protocol.

Total RNA was extracted 24 h post-transfection using TRIzol™ Reagent (Invitrogen, Thermo Fisher Scientific, Waltham, MA, USA), followed by DNase treatment with TURBO™ DNase (Invitrogen, Thermo Fisher Scientific, Waltham, MA, USA).

### Sephadex bead preparation

A total of 250 mg of Sephadex G-100 beads (#S6147; Sigma-Aldrich, St. Louis, MO, USA) was swollen in 10 mL of bead storage buffer (50 mM HEPES, pH 7.5, 100 mM NaCl, 1 mM MgCl₂, and 0.05% Tween-20) and incubated at room temperature (25°C) for 72 h before use. The beads were washed three times by centrifugation at 1000 × g for 30 seconds using an Ultrafree® Centrifugal Filter (pore size: 0.22 µm, PVDF membrane, 0.5 mL sample volume; Millipore Sigma, Burlington, MA, USA).

Beads were equilibrated by incubating them in binding buffer (50 mM HEPES, pH 7.5, 100 mM NaCl, 1 mM MgCl₂, 20 µg/mL yeast tRNA, and 0.025 U/µL RNase OUT) before RNA purification.

### RNA binding and elution

Before binding, RNA samples were denatured at 65°C for 10 min and allowed to refold at room temperature for 5 min.

For RNA binding kinetics and optimization, FS2-tagged RNA (10–100 pmol) was incubated with 50 µL of Sephadex beads at 4°C for 0–240 min with gentle rotation. Unbound RNA was collected from the flow-through, and beads were washed three times with binding buffer (50 mM HEPES, pH 7.5, 100 mM NaCl, 1 mM MgCl₂, 20 µg/mL yeast tRNA, and 0.025 U/µL RNase OUT). To evaluate the effect of Mg²⁺ concentration, binding experiments were performed with varying MgCl₂ concentrations (0, 0.5, 1, 5, and 10 mM) in the presence of 100 mM NaCl. To determine optimal stringency conditions, additional experiments were performed using varying NaCl concentrations (100–800 mM) in the presence of 1 mM MgCl₂.

For elution, Sephadex-bound RNA was incubated at 50°C with elution buffer containing 50 mM HEPES and 10–50 mM EDTA for 10 min, and the eluate was collected for downstream analysis. For competitive elution, Sephadex-bound RNA was incubated with binding buffer supplemented with 50 mg/mL dextran from *Leuconostoc mesenteroides* (average molecular weight 9000–11 000; D9260, Sigma-Aldrich).

Eluted RNA was assessed using denaturing PAGE (Novex™ 6% TBE-Urea Gels; Invitrogen, Thermo Fisher Scientific, Waltham, MA, USA) and analyzed using the Agilent TapeStation system (Agilent, Santa Clara, CA, USA). Band intensities were quantified by densitometric analysis using ImageJ and normalized to the maximum signal within each replicate.

### TapeStation-based microfluidic electrophoresis analysis

RNA samples were analyzed using an Agilent 4150 TapeStation system with RNA ScreenTape and RNA ScreenTape reagents (Agilent, Santa Clara, CA, USA), according to the manufacturer’s instructions. TapeStation analysis is based on microfluidic electrophoretic separation, followed by ladder-based alignment and software-assisted sizing.

Because sample matrix composition can affect electrophoretic migration and ladder-based sizing assessment, apparent migration differences in TapeStation analysis were interpreted cautiously, particularly for samples containing EDTA or detergent-containing buffers. Agilent’s technical guidance notes that excessive salt in the sample matrix can slow sample migration, interfere with ladder alignment, and lead to inaccurate sizing assessment [11]. In many cases, the samples used in this study exceeded the maximum buffer concentrations recommended in the TapeStation specifications, which comprise 200 mM Tris, 20 mM EDTA, and 50 mM NaCl. For this reason, the mobility and intensity of bands can be affected.

### RT–qPCR analysis

RNA from input and purification fractions was purified and reverse-transcribed using random hexamers and gene-specific primers according to the manufacturer’s instructions. For each comparison, equal volumes of the processed input and purification fractions were used for reverse transcription. Quantitative PCR was performed using primer sets specific for the FS2/FS4 reporter RNA and *GAPDH*, with reactions performed in technical triplicate. Relative RNA abundance was calculated using the ΔCt or ΔΔCt method. Recovery was calculated by comparing the reporter RNA level in the elution fraction to the corresponding input sample. Fold enrichment was calculated by normalizing the reporter RNA signal to *GAPDH* and comparing elution fractions to input.

### Western blot analysis

Western blotting was performed to assess protein interactions with FS2-tagged RNA constructs. Protein samples were separated using 4–12% Bis-Tris polyacrylamide gels (Invitrogen, Thermo Fisher Scientific, Waltham, MA, USA) and transferred onto polyvinylidene difluoride (PVDF) membranes (Trans-Blot Turbo Mini, 0.2 µm; Bio-Rad, Hercules, CA, USA). Membranes were blocked with 5% bovine serum albumin (BSA) in Tris-buffered saline containing 0.1% Tween-20 (TBST) for 1 h at room temperature to prevent non-specific binding.

Primary antibody incubations were conducted overnight at 4°C, using the following antibodies: anti-FLAG (1:2000; Sigma-Aldrich, St. Louis, MO, USA) for MS2-MCP purification, anti-YTHDF2 (1:1000; Proteintech, Rosemont, IL, USA) for endogenous RNA-protein pulldown, and anti-GAPDH (1:5000; Cell Signaling Technology, Danvers, MA, USA) as a loading control. Membranes were subsequently washed five times with TBST and incubated for 1 h at room temperature with horseradish peroxidase (HRP)-conjugated secondary antibodies.

Detection of protein bands was performed using the ECL Western blot substrate (Cytiva, Marlborough, MA, USA), and chemiluminescent signals were visualized using the ChemiDoc MP Imaging System (Bio-Rad, Hercules, CA, USA).

### RNA–protein complex purification using FS2-tagged RNA

To study RNA–protein interactions, two experimental approaches were employed.

For the MS2-MCP (MS2 coat protein) pulldown, HEK293T cells were co-transfected with plasmids encoding FS2-tagged MS2 RNA and FLAG-tagged MCP. The nucleotide and amino acid sequences of FLAG–MCP are provided in Supplementary Table S2. After 48 h, cells were harvested and lysed in lysis buffer containing 50 mM HEPES, 100 mM NaCl, 1 mM MgCl₂, and 1% NP-40, pH 7.5.

For the endogenous YTHDF2 pulldown, wild-type and *METTL3* knockout mouse embryonic stem cells (mESCs) were transfected with FS2-tagged DRACH RNA. After 48 h, cells were harvested and lysed using the same lysis buffer.

In both cases, cell lysates were incubated with Sephadex beads at 4°C for 100 min, followed by five washes in binding buffer. Protein–RNA complexes were then eluted and analyzed by Western blot, as described above.

### Data analysis

All experiments were performed with at least three independent biological replicates (*n* = 3) unless otherwise stated.

## Results

### The D8 aptamer efficiently binds Sephadex beads

**Figure 1.**
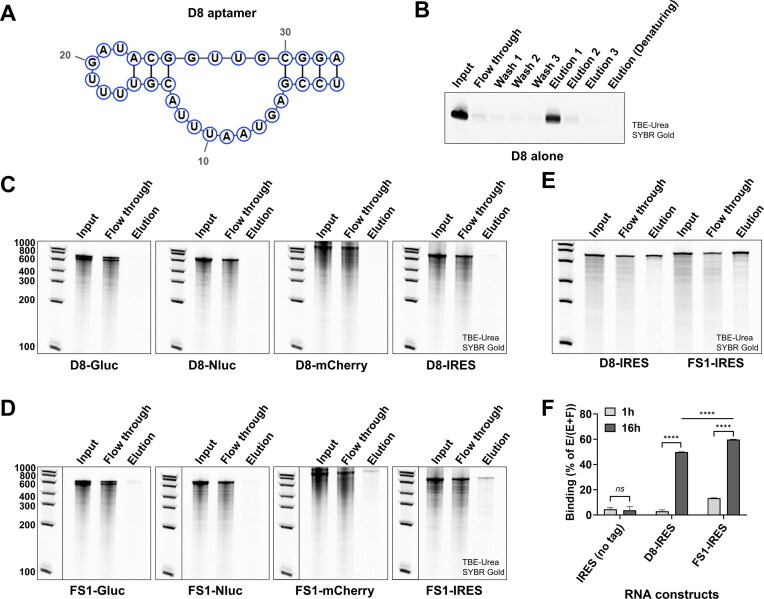
Structural characterization and binding efficiency of the D8 Sephadex-binding aptamer in RNA affinity purification. (**A**) mFold-predicted secondary structure of the D8 aptamer, highlighting essential motifs required for Sephadex binding. (**B**) Binding efficiency of the D8 aptamer alone was analyzed using TBE-urea gel electrophoresis following Sephadex purification. *In vitro* transcribed D8 RNA was incubated with Sephadex beads, and bound RNA was sequentially eluted using dextran buffer (50 mg/mL, three rounds) followed by a final denaturing elution with urea buffer (8 M urea and 5 mM EDTA). (**C**) *In vitro* binding efficiency of D8-tagged mRNAs (Nanoluc, Gaussia luciferase, mCherry, and IRES) was assessed by incubating *in vitro*-transcribed RNAs with Sephadex beads, followed by analysis via TBE-urea gel electrophoresis. Results indicate that larger RNA constructs exhibit reduced binding efficiency, suggesting potential folding interference. (**D**) Effect of F30 scaffolding on D8 function was evaluated, showing increased RNA retention compared to unscaffolded D8, indicating enhanced aptamer folding and binding accessibility. (**E**) Comparative analysis of D8-tagged IRES and FS1-tagged IRES constructs with extended binding times (16 h). (**F**) Quantification of RNA bound after extended incubation. The bar graph shows the percentage of input RNA retained by Sephadex beads for D8-tagged IRES and FS1-tagged IRES constructs, calculated as RNA levels in the eluate / (flow-through + eluate), based on densitometric analysis of band intensities using ImageJ. After 1 h, 3% of D8-IRES and 13.3% of FS1-IRES were bound. Extending the binding time to 16 h substantially increased bound RNA to ∼50% for D8-IRES and ∼60% for FS1-IRES. Prolonged incubation (16 h) significantly improved RNA binding, particularly for FS1-IRES, demonstrating that extended binding durations mitigate misfolding effects and enhance RNA sequestration by Sephadex beads. As a control, untagged IRES RNA showed no detectable signal in the elution fraction under the same conditions, indicating that RNA binding to Sephadex is dependent on the presence of the D8 or FS1 tag. Statistical significance between the 1 and 16 h incubation conditions for each RNA construct was determined by two-way ANOVA followed by Šídák’s multiple comparisons test. Statistical significance between D8-IRES and FS1-IRES at 16 h was determined using an unpaired two-tailed t-test. Data are shown as mean ± SD from three independent experiments. *ns*, not significant; ****P < 0.0001.

The D8 aptamer is a 33-nucleotide RNA (Fig. 1A) that was previously identified through SELEX and exhibits high specificity for Sephadex G-100 beads [8]. Sephadex G-100 is primarily composed of dextran B512, which consists mainly of α-1,6-linked glucose residues with a smaller fraction of α-1,3 linkages. The D8 aptamer shows no affinity for smaller oligosaccharides such as isomaltose, isomaltotriose, or isomaltotetraose.

To evaluate whether the D8 aptamer can function as an affinity tag, we first tested the D8 aptamer alone, prepared by *in vitro* transcription. The D8 aptamer displayed strong binding to Sephadex beads, based on the detection of only minimal levels of the D8 RNA in the flow-through. Sephadex-bound RNA was efficiently eluted using a protocol involving sequential elution with dextran buffer (binding buffer supplemented with 50 mg/mL dextran), consistent with competitive displacement of the aptamer from Sephadex (Fig. 1B). These results confirm the aptamer’s ability to bind Sephadex and demonstrate its potential utility as an RNA affinity tag.

We next asked if D8 can function as an affinity tag when appended to another RNA. To test this, we inserted the D8 aptamer sequence on the 3′ ends of four RNAs: Nanoluc (Nluc) mRNA (516 nt), Gaussia luciferase (Gluc) mRNA (558 nt), mCherry mRNA (711 nt), and IRES mRNA (609 nt). Each RNA was prepared by *in vitro* transcription, and 100 pmol of each was incubated with Sephadex beads for 1 h, followed by washing and elution with urea buffer. Surprisingly, all the tagged RNAs appeared in the flow-through, with none detectable in the eluate (Fig. 1C).

Overall, these data suggest that the D8 aptamer can in principle bind Sephadex and be used for purification. However, in the context of a larger RNA, its folding or ability to bind the Sephadex is impaired.

### Enhancing D8 folding slightly improves the function of D8 as an affinity tag

We hypothesized that the D8 aptamer may not fold properly when surrounded by adjacent RNA sequences, which might cause misfolding of the D8 aptamer. This would prevent D8 from adopting its optimal conformation, reducing its interaction with Sephadex beads.

We therefore tested the F30 scaffold, an RNA three-way junction derived from bacteriophage phi29, which forms part of the packaging RNA [12–14]. We previously modified phi29 so that sequences matching the RNA polymerase III termination element were removed, which allowed it to be expressed in mammalian cells, and named the new variant F30 [15]. We showed that insertion of aptamers into either stem-loop structure of F30 markedly increases the folding of these aptamers and insulates the RNA aptamer from inhibitory folding effects from neighboring sequences. For example, F30 enhanced the folding of the fluorogenic aptamer Broccoli when inserted as a tandem repeat in mRNAs [16].

To determine if the F30 scaffold could improve D8 function, we tagged the 3′ ends of four RNA sequences (Nanoluc, Gaussia luciferase, mCherry, and IRES) with an FS1 sequence, comprising D8 inserted into the left arm of F30. We then repeated the Sephadex-binding experiments. As shown in Fig. 1D, the F30 scaffold modestly improved D8 folding, leading to a small but measurable increase in RNA binding. After 1 h of binding, the eluted FS1-tagged RNA comprised ∼13.3% of the input for the FS1 construct, compared to only 3% for the unscaffolded D8 construct (Fig. 1D). While this improvement highlights the potential of scaffolding to enhance aptamer folding, the majority of the tagged RNA still remained in the flow-through, indicating that further improvements were needed.

We next hypothesized that longer binding times could improve binding by providing the aptamer more time to bind the Sephadex. Unlike small RNAs, which can rotate rapidly in solution and adopt an orientation that allows binding to Sephadex, larger RNAs may require more time for achieving an orientation needed for binding. To test this, we repeated the binding experiments with an extended incubation time of 16 h. This longer incubation time substantially enhanced RNA binding across multiple RNA contexts, including Nluc, Gluc, mCherry, and IRES RNAs (Supplementary Fig. S1). Specifically, the eluted RNA for the D8-IRES construct increased from 3% (after 1 h) to 50% of the input, while the FS1-IRES construct improved from 13.3% to 60% of the input (Fig. 1E and F). This substantial improvement suggests that extended binding times are needed for larger D8-containing RNAs to bind, and that the F30 scaffold is needed for improved D8 folding. To determine whether the 16 h incubation leads to nonspecific binding, untagged IRES RNA was tested under the same conditions. However, no detectable IRES RNA was observed in the elution fraction, indicating that RNA binding to Sephadex is dependent on the presence of the D8 or FS1 tag (Supplementary Fig. S2).

### Design of FS2, a high-affinity Sephadex-binding tag

**Figure 2.**
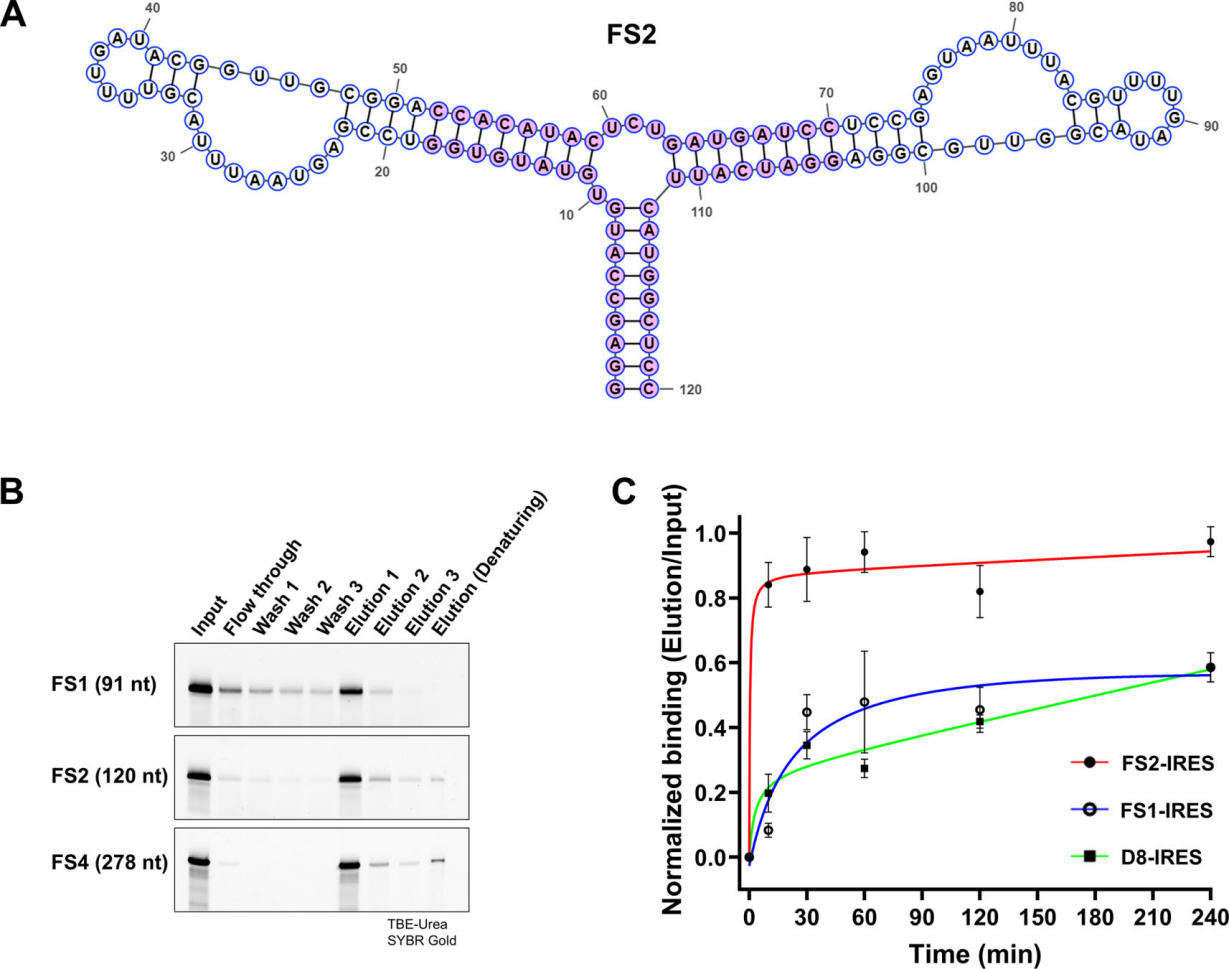
Design and functional validation of FS2, a high-affinity Sephadex-binding RNA tag. (**A**) Predicted secondary structure of FS2, incorporating two D8 aptamers within the F30 scaffold to enhance binding avidity and improve RNA purification efficiency. The F30 scaffold regions are highlighted. (**B**) Comparison of RNA binding and elution among FS1, FS2, and FS4 constructs, analyzed by TBE-urea gel following Sephadex purification. FS2 and FS4 exhibit higher retention and elution efficiency, confirming that multivalent aptamer presentation improves RNA binding. (**C**) Time-course binding analysis of D8, FS1, and FS2 with IRES RNA, demonstrating enhanced binding kinetics over a 0–240 min incubation period. FS2 achieves greater RNA binding than D8 and FS1, indicating improved stability and efficiency in RNA purification.

Although our results showed that F30 can enhance the Sephadex-binding ability of D8, we wanted to increase the amount of RNA that binds and the rate of binding. To enhance binding affinity, we adopted an avidity approach by incorporating multiple D8 aptamers within the F30 scaffold. The F30 scaffold, with its dual-arm configuration, can accommodate one D8 aptamer in each arm. We designated the single-aptamer construct as FS1, and the dual-aptamer construct as FS2 (Fig. 2A). To further increase avidity, we also designed a tandem construct consisting of two sequential FS2 units, resulting in a four-aptamer configuration designated FS4 (Supplementary Fig. S3).

We tested the binding of the FS1, FS2, and FS4 RNA tags, without additional RNA sequences (100 pmol of RNA), to Sephadex. While shorter aptamers such as D8 can exhibit efficient binding in isolation due to more favorable folding, we wanted to confirm that embedding the aptamer in the F30 scaffold did not impair the ability of D8 to bind Sephadex. FS1 showed the most RNA in the flow-through and washes. However, FS2 showed much less RNA in the flow-through. FS4 showed even less RNA in the flow-through (Fig. 2B). To assess the binding, we also eluted the RNAs from Sephadex. Elution was carried out with three sequential washes in dextran buffer (binding buffer supplemented with 50 mg/mL dextran) followed by urea buffer (8 M urea, 5 mM EDTA), and a final boiling step at 95°C for 5 min to ensure that any remaining RNA was eluted from the Sephadex beads. Under these stringent elution conditions, FS1 released little RNA, consistent with weak initial binding. In contrast, both FS2 and FS4 retained substantial RNA even after sequential washes with dextran and urea buffer, and a fraction remained bound until boiling at 95°C. These results confirm that FS2 and FS4 form exceptionally stable interactions with Sephadex. Overall, FS2 and FS4 bind more efficiently to Sephadex. However, since the improvement of FS4 was fairly small relative to FS2, and because of the large size (278 nt) of FS4, we focused on the smaller FS2 design.

Our previous results suggested that FS1 needed longer binding times to achieve binding (see Fig. 1E and F). To quantitatively assess whether FS2 needed shorter binding times, we compared D8 and FS1- and FS2-tagged IRES RNA with Sephadex beads for various durations (0–240 min), followed by elution with urea buffer (Fig. 2C). In this analysis, binding was quantified as the ratio of RNA signal in the elution fraction relative to the input (E/I) to monitor the accumulation of bound RNA over time. This differs from the analysis used in Fig. 1F, where binding efficiency was calculated as E/(F + E), reflecting the fraction of RNA bound relative to unbound RNA. Because these metrics capture different aspects of binding behavior, the absolute values are not directly comparable between the two analyses. The FS2-tagged IRES construct achieved over 80% binding within 10 min, increasing to 95% after 1 h. In comparison, FS1-tagged IRES showed slower kinetics and lower binding, while the unscaffolded D8-tagged IRES construct demonstrated minimal levels of retained RNA on the Sephadex in these conditions. These data suggest that FS2 allows for faster binding to Sephadex beads.

Remarkably, 84% of FS2-tagged IRES RNA was bound after just 10 min of incubation, increasing to 88% by 30 min and further reaching 95% after 1 h (Fig. 2C). These results demonstrated that the FS2 construct achieves rapid and efficient binding, making it a high-performance affinity tag for RNA purification.

### Optimization of the binding conditions for FS2-tagged RNAs

Our initial experiments led to the identification of FS2 as the best affinity tag. We next sought to systematically optimize the binding conditions for FS2-tagged IRES RNA to establish a robust and reproducible protocol for RNA purification.

#### Establishing the optimal RNA loading capacity for FS2-tagged RNAs

Determining the optimal RNA loading capacity is essential to maximize binding efficiency and ensure consistent performance during purification. Overloading Sephadex beads can lead to binding saturation, resulting in excess RNA in the flow-through and inaccurate recovery. Conversely, suboptimal RNA input may underutilize the binding capacity of the beads, reducing the overall yield. By identifying the optimal loading capacity, we can establish conditions that provide reproducible results and maintain the integrity of RNA purification workflows.

**Figure 3.**
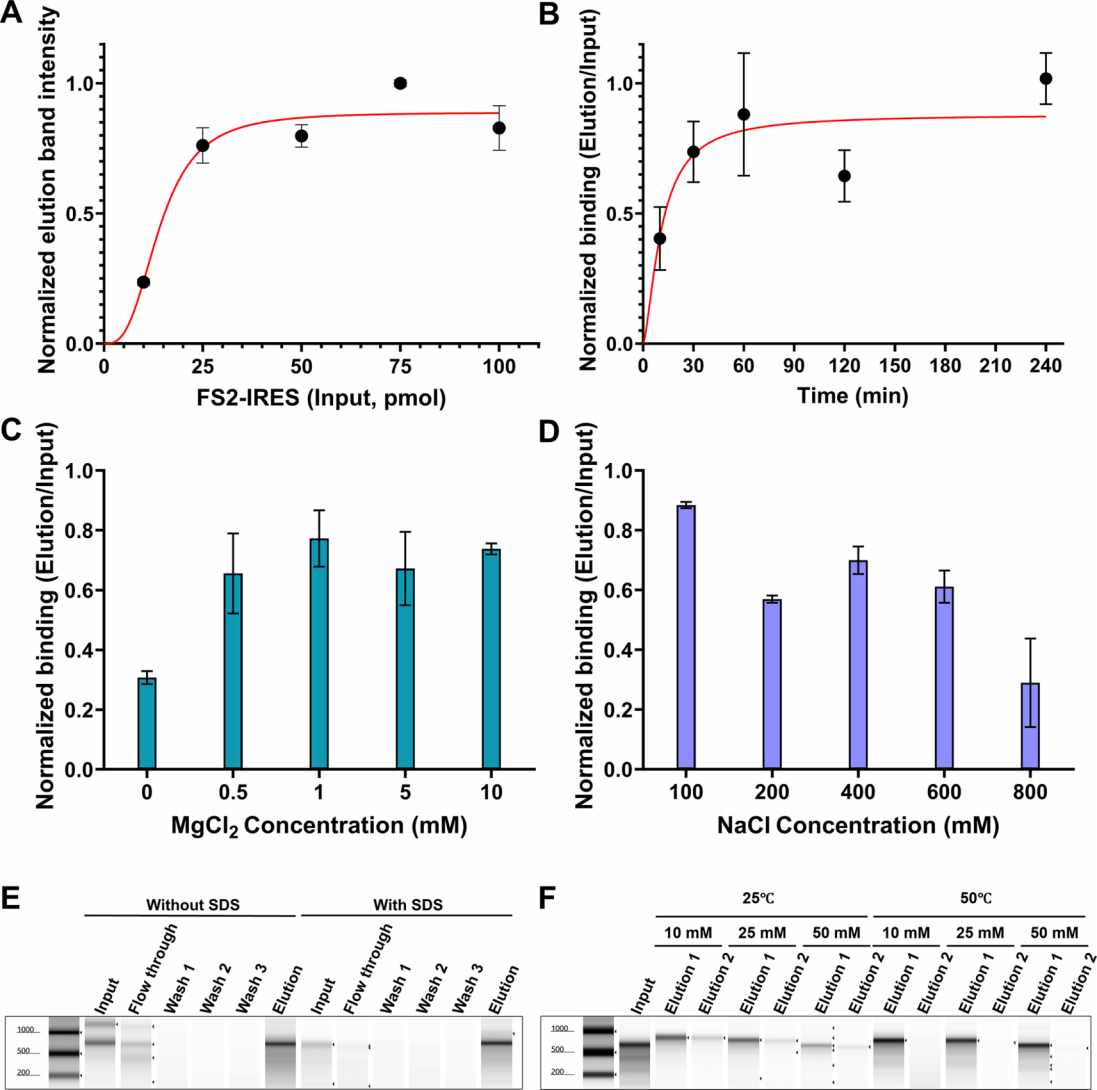
Systematic optimization of FS2-mediated RNA purification conditions. (**A**) Determination of optimal RNA loading conditions. Different amounts of FS2-tagged RNA (10–100 pmol) were incubated with Sephadex beads, and relative elution signal was quantified by densitometric analysis, identifying ∼25 pmol as an optimal input amount where the elution signal approaches a plateau. Elution band intensities were quantified using ImageJ and normalized within each replicate. (**B**) Binding kinetics of FS2-tagged RNA, showing that maximum retention on Sephadex is achieved within 60 min, indicating rapid and efficient binding. (**C**) Effect of Mg²⁺ concentration on FS2-mediated RNA purification. RNA binding was assessed across increasing MgCl₂ concentrations in the presence of 100 mM NaCl. While a low concentration (0.5 mM) is required for efficient binding, increasing Mg²⁺ beyond this threshold does not improve output, suggesting FS2 functions optimally under minimal Mg²⁺ conditions. (**D**) Optimization of NaCl concentration for washing conditions. The retained amount of FS2-tagged IRES RNA was measured following washing with increasing NaCl concentrations (100–800 mM) in the presence of 1 mM MgCl₂. Binding was largely maintained up to 600 mM NaCl but decreased at higher concentrations, indicating disruption of FS2–Sephadex interactions at excessive ionic strength. Based on these results, 100 mM NaCl was selected for standard binding, and concentrations up to 600 mM were considered suitable for stringent washing without compromising RNA retention. (**E**) Compatibility of FS2-mediated RNA binding with SDS. FS2-tagged IRES RNA was incubated with Sephadex beads in binding buffer with or without 1% SDS at 4°C for 20 h. Comparable RNA binding was observed under both conditions, indicating that SDS does not affect FS2-mediated RNA binding. Reduced signal in the SDS-treated input and wash fractions is likely due to detergent effects on RNA detection, particularly in microfluidic electrophoresis systems. (**F**) Optimization of elution conditions, revealing that a combination of elevated temperature (50°C) and 10 mM EDTA maximizes RNA release from Sephadex beads, providing a streamlined and effective elution strategy. It should be noted that the altered migration of eluted RNA bands seen here is likely due to differences in sample buffer composition during TapeStation analysis, which can affect electrophoretic migration and ladder-based sizing assessment [11], as described in Materials and methods.

To establish the appropriate amount of input RNA, we tested increasing amounts of FS2-tagged IRES RNA (10–100 pmol) with 50 µL of Sephadex beads at 4°C for 60 min. The RNA distribution across input, flowthrough, and elution fractions was analyzed using a microfluidic capillary electrophoresis system (Agilent TapeStation), followed by densitometric quantification of band intensities using ImageJ (Fig. 3A). Elution band intensities were normalized within each replicate. The normalized elution band intensity increased with increasing RNA input but approached a plateau at ∼25 pmol of FS2-tagged RNA, while excess RNA increasingly appeared in the flow-through fraction. These findings suggest that ∼25 pmol RNA per 50 µL of Sephadex beads represents a practical loading amount for efficient and reproducible RNA purification under these conditions.

#### Binding kinetics of FS2-tagged RNA: determining the optimal incubation time

Next, we investigated the binding kinetics of FS2-tagged RNA to Sephadex beads to determine the time required for optimal interaction. Binding kinetics are critical for developing efficient protocols and ensuring reliable RNA–Sephadex interactions.

Using the previously established loading capacity of 25 pmol of FS2-tagged IRES RNA, binding assays were performed over incubation times ranging from 0 to 240 min (Fig. 3B). The results showed that RNA binding reached saturation within 60 min, with no significant increase in RNA bound observed beyond this time point. These findings indicate that a 60-min incubation is sufficient to achieve optimal binding, providing a streamlined and standardized protocol for efficient RNA purification.

#### Impact of magnesium on FS2-tagged IRES binding efficiency

Magnesium ions (Mg²⁺) are known to stabilize RNA secondary and tertiary structures, which are critical for maintaining binding efficiency in many RNA-based affinity systems. To determine whether Mg²⁺ is essential for FS2-mediated RNA binding, we tested binding efficiency across a range of Mg²⁺ concentrations (0, 0.5, 1, 5, and 10 mM). FS2-tagged IRES RNA (25 pmol) was incubated with 50 µL of Sephadex beads at 4°C for 60 min, and RNA bound to Sephadex was quantified (Fig. 3C).

In the absence of Mg²⁺ (0 mM), binding was significantly impaired, with most RNA detected in the flow-through, suggesting that proper RNA folding is required for FS2 function. However, 0.5 mM Mg²⁺ substantially increased binding, with slight further improvements at 1 mM, but no detectable improvements at higher Mg²⁺ concentrations up to 10 mM. These results indicate that while a minimal amount of Mg²⁺ is necessary for RNA folding and effective FS2 binding, higher Mg²⁺ concentrations do not further enhance binding efficiency. Thus, 0.5–1 mM Mg²⁺ appears sufficient for optimal RNA binding, simplifying buffer formulation while maintaining robust purification performance.

#### Optimizing NaCl concentration for stringent washing conditions

Salt concentration plays a critical role in RNA purification by reducing non-specific interactions while preserving the intended RNA-Sephadex binding. In practical applications, elevated salt is often used to reduce background binding from cellular RNAs, but excessive ionic strength can also weaken the desired RNA–ligand interaction. We therefore sought to determine how resistant the FS2–Sephadex interaction is to increasing NaCl concentrations.

We tested NaCl concentrations ranging from 100 to 800 mM and measured the amount of FS2-tagged IRES RNA retained on Sephadex (Fig. 3D). Retention remained high up to 600 mM NaCl but declined at higher concentrations, suggesting that excessive ionic strength disrupts FS2–Sephadex binding. Based on these results, we used 100 mM NaCl for standard binding conditions and identified up to 600 mM NaCl as a suitable range for stringent washing without compromising binding of FS2 to Sephadex.

This analysis provides a benchmark for designing washing conditions that maintain high yields of FS2-tagged RNA while allowing flexibility for salt-based stringency in more complex RNA mixtures.

#### SDS does not interfere with FS2-tagged RNA binding

Sodium dodecyl sulfate (SDS) is widely used in RNA purification workflows to prevent RNA aggregation, inhibit RNase, and minimize unwanted interactions with proteins or other RNA molecules. However, its potential impact on the ability of FS2-tagged RNA to bind to Sephadex is unclear.

To assess whether SDS influences the purification process, FS2-tagged IRES RNA was incubated with Sephadex beads in binding buffers with or without 1% SDS at 4°C for 20 h (Fig. 3E). The results showed that SDS had no detectable effect on FS2-tagged IRES binding efficiency, as the amount of RNA bound to Sephadex remained comparable between conditions with and without SDS. Since SDS is known to reduce RNase activity, its inclusion could help maintain RNA integrity during extended incubations.

These findings confirm that SDS can be incorporated into FS2-tag-based purification workflows without interfering with RNA binding. Its inclusion may be beneficial for reducing non-specific interactions and for denaturing proteins such as RNases, which are particularly problematic in protocols requiring prolonged handling. SDS can be removed by including SDS-free bead-washing steps prior to elution.

Efficient RNA purification under detergent-containing lysis conditions is important for applications involving mammalian cell extracts. To further evaluate the robustness of FS2-mediated purification, we tested RNA purification in the presence of 1 × RIPA buffer, which contained a final concentration of 0.1% SDS, 0.5% sodium deoxycholate, and 1% NP-40 (or Triton X-100). Since RIPA buffer contains EDTA, which would chelate magnesium needed for the folding of the D8 aptamer, the MgCl₂ concentration was increased to 10 mM to compensate for EDTA present in RIPA. Under these conditions, FS2-mediated purification remained effective, with clear recovery of FS2-tagged RNA in the elution fraction (Supplementary Fig. S4). These results indicate that FS2 is compatible with detergent-containing lysis conditions.

#### Optimizing elution conditions: the combined effect of temperature and EDTA

Efficient elution conditions are critical for maximizing RNA recovery while maintaining structural integrity for downstream applications. To optimize FS2-tagged RNA elution, we examined the effects of temperature and EDTA concentration on FS2-tagged IRES RNA release from Sephadex beads. Binding reactions were conducted under optimized conditions, and elutions were performed using buffers containing 50 mM HEPES with varying EDTA concentrations (10 mM, 25 mM, and 50 mM) at either 25°C or 50°C (Fig. 3F).

At 25°C, all tested EDTA concentrations resulted in incomplete RNA elution, with residual RNA remaining bound to the beads. In contrast, at 50°C, RNA elution was markedly improved, with ≥10 mM EDTA effectively eluting most FS2-tagged IRES RNA in a single step. To verify completeness of elution, we performed a second elution under identical conditions. Negligible RNA was observed in this step, indicating that the combination of elevated temperature and EDTA was sufficient for near-complete release in the initial elution.

These results demonstrate that combining an elevated temperature (50°C) with sufficient EDTA concentration (≥10 mM) enhances RNA elution efficiency, providing optimized conditions for FS2-tagged RNA purification.

### Affinity purification of overexpressed *MYC* mRNA using the FS2-tag

**Figure 4.**
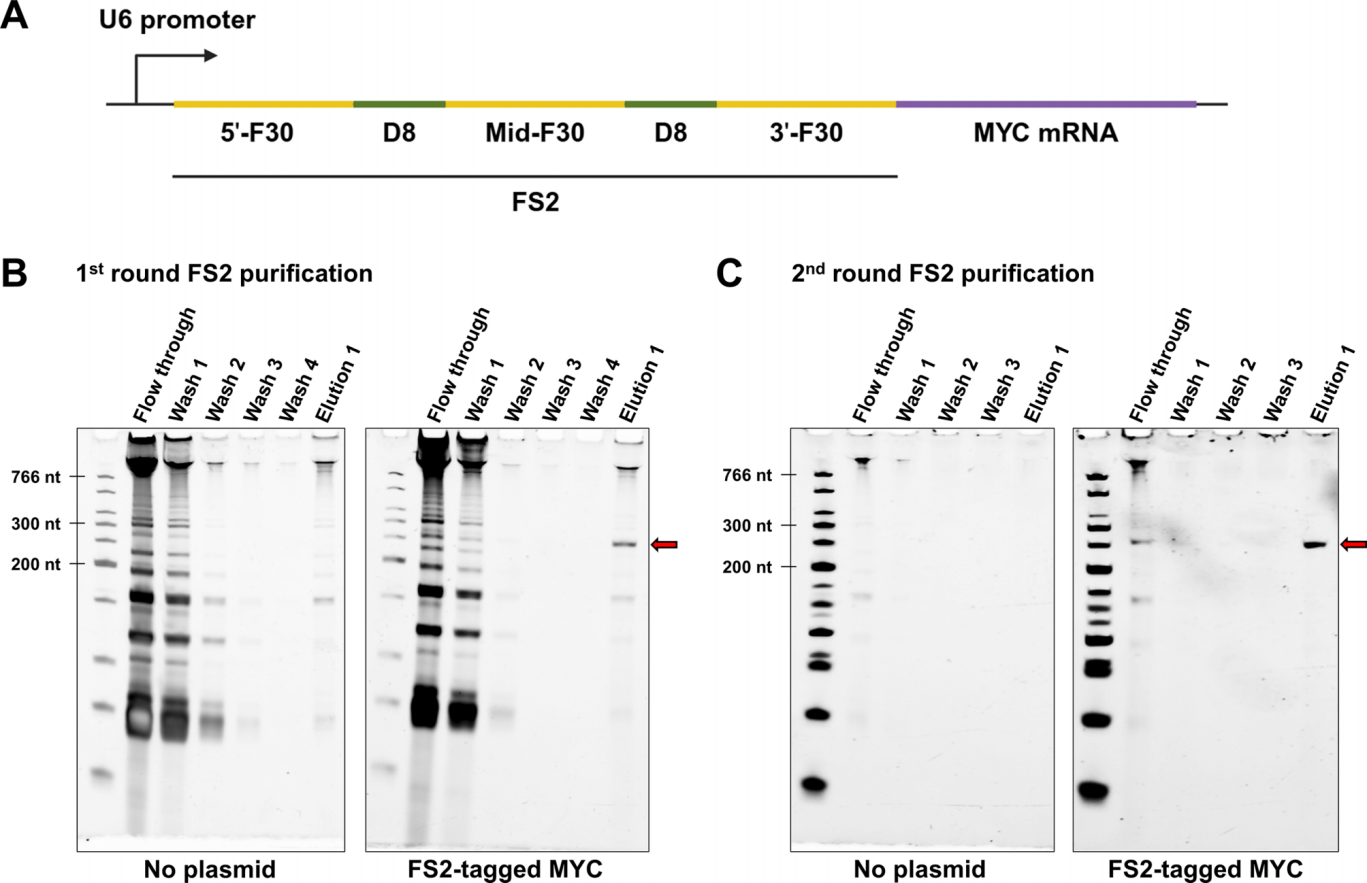
Affinity purification of FS2-tagged *MYC* mRNA fragment from HEK293T cells. (**A**) Schematic representation of the FS2-tagged *MYC* RNA construct designed for affinity purification. (**B**) TBE-urea gel analysis demonstrating specific enrichment of the FS2-tagged *MYC* RNA fragment after 1-h incubation, with minimal background contamination observed in control samples. (**C**) Two-round purification workflow, confirming high specificity and purity of FS2-tagged *MYC* RNA, validating the robustness of FS2 in cellular RNA purification. The schematic illustration in panel A was created in BioRender. Jaffrey, S. R. (2026) https://BioRender.com/u5keknv.

We next sought to test the affinity purification of tagged RNAs from cellular extracts. To evaluate whether the FS2 tag can facilitate the selective purification of an RNA from cellular extracts, we designed an FS2-tagged construct containing a 51 nt-long region of the *MYC* mRNA (genomic location: chr8:127 738 635–127 738 685 [hg38]). In contrast to the examples above, in which FS2 was positioned at the 3′ end of the RNA constructs, we placed the FS2 tag at the 5′ end of a *MYC* mRNA fragment to evaluate whether FS2-mediated purification is compatible with alternative tag positions. The RNA was expressed using the U6 promoter to drive RNA expression using RNA Polymerase III (Fig. 4A). HEK293T cells were transfected with this construct, and total RNA was extracted 24 h post-transfection. FS2-tagged *MYC* RNA was then purified using Sephadex-based affinity purification, followed by elution and TBE-urea gel analysis.

The results demonstrated that FS2-tagged *MYC* RNA was efficiently isolated from transfected HEK293T cells, with a few other bands, representing contamination from endogenous RNAs (Fig. 4B). Densitometry of the eluted sample indicated that the FS2-tagged *MYC* RNA band accounted for 29.9 ± 4.6% of the RNA in the eluate after the first round of purification. In contrast, eluates from untransfected cells lacked a band at the expected size, confirming that isolation of the *MYC* RNA was dependent on FS2 tagging.

To further improve enrichment of the target RNA, we performed a second round of purification. After the first round, the eluate was subjected to ethanol precipitation to remove EDTA, salts, and small contaminants. The purified RNA was then reapplied to Sephadex-based affinity purification for a second round of binding, washing, and elution under standard conditions. This two-step protocol substantially reduced contaminating RNAs, with densitometry showing that FS2-tagged *MYC* RNA accounted for 89.9 ± 3.7% of the RNA in the eluate after the second round (Fig. 4C). These data indicate that one round yields a highly enriched RNA from cellular extracts, and that target RNA enrichment can be further improved using a second round of RNA purification.

As an additional test, we sought to purify a circular RNA. We expressed the *MYC* mRNA region in the Tornado expression plasmid [10], which expresses RNAs flanked with twister ribozymes, resulting in subsequent end-to-end ligation with the endogenous RNA ligase RtcB. The construct also contained an FS2 tag for affinity purification. For comparison, we examined both linear and circular FS2-tagged *MYC* RNAs. The circular form yielded a higher signal compared to the linear form (Supplementary Fig. S5). This difference likely reflects increased expression and/or stability of circular RNA compared to the linear form. Overall, FS2 affinity purification is fully compatible with both linear and circular RNA constructs. These findings also show the versatility of FS2 tagging for isolating specific RNAs directly from mammalian cells, offering a robust approach for tagged transcript purification in cellular contexts.

To directly compare FS2 with the original D8 aptamer in a cellular circular RNA context, we generated circular *MYC* RNA constructs containing either the D8 aptamer alone or the FS2 tag and subjected them to Sephadex-based purification under identical conditions. While FS2-tagged circular *MYC* RNA was readily detected in the elution fraction, the D8-tagged construct showed little to no detectable recovery (Supplementary Fig. S6). These results indicate that incorporation into the FS2 scaffold substantially improves the ability of D8-containing circular RNAs to undergo efficient purification in mammalian cells.

Consistent with this, we further demonstrate that FS2 enables specific purification of a 1.6 kb m^6^A reporter mRNA previously described [17], supporting its applicability to longer and biologically relevant RNA targets (Supplementary Fig. S7). RT–qPCR analysis further confirmed specific recovery of the FS2-tagged reporter mRNA in purified eluates, whereas only background-level amplification was observed in untransfected control (Supplementary Fig. S7B). In addition, RT–qPCR analysis estimated an apparent recovery of 58.4 ± 4.4% following FS2-mediated purification, indicating efficient recovery of the long mRNA target (Supplementary Fig. S7C). These results further support the applicability of FS2-mediated purification to long mRNA targets in mammalian cells.

Next, we asked whether multivalent FS2 could be used to enrich a low-abundance mRNA reporter. We constructed an FS2-IRES-EGFP reporter and destabilized the reporter transcript using a 5′ self-cleaving ribozyme, which has been shown to reduce reporter RNA expression [18, 19]. After transfection, the reporter was expressed approximately eight-fold lower than *GAPDH*, placing it within the range expected for many endogenous transcripts. However, the recovery of this low-abundance FS2 reporter was low.

We therefore asked whether increasing the valency of the Sephadex-binding aptamer could improve purification of this low-abundance reporter. To test this, we replaced FS2 with FS4 and repeated the transfection and Sephadex enrichment protocol. As with the FS2-containing reporter, the FS4-IRES-EGFP reporter was also expressed approximately eight-fold lower than *GAPDH* in the input sample (Supplementary Fig. S8A). After Sephadex purification, *GAPDH* mRNA was near the limit of detection in the first elution fraction based on RT-qPCR, whereas the FS4 reporter was readily detected (Supplementary Fig. S8B). After normalizing for the total sample volumes used for RT-qPCR, we estimated that Sephadex purification recovered 14.0 ± 0.4% of the total FS4 reporter RNA and enriched the FS4 reporter by approximately 79 000-fold relative to *GAPDH* (Supplementary Fig. S8C and D). Together, these data demonstrate that increasing FS aptamer valency enables purification and strong enrichment of a low-abundance mRNA reporter expressed at a level comparable to many endogenous transcripts.

### FS2 enables RNA–protein complex purification in exogenous and endogenous systems

#### Validating FS2 for RNA–protein interaction studies using the MS2-MCP system

**Figure 5.**
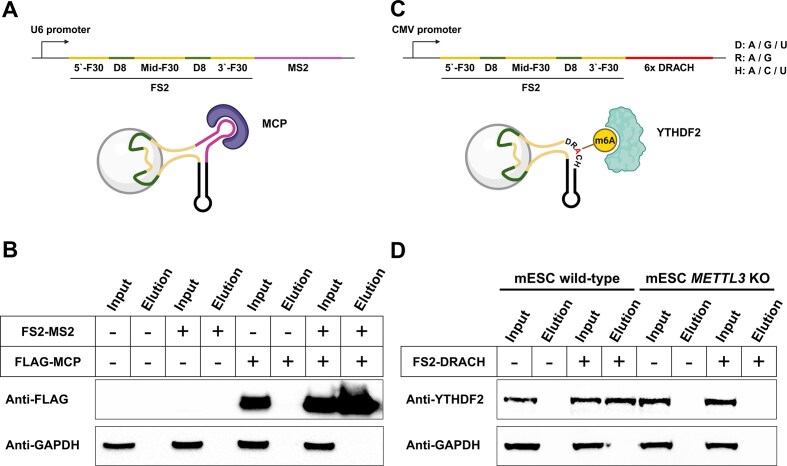
FS2-mediated pulldown application of RNA-protein complexes for RNA interactome studies. (**A**) Schematic illustration of the FS2-tagged MS2 construct, in which the FS2 tag was incorporated at the 5′ end of MS2 RNA and expressed under the U6 promoter. (**B**) Validation of FS2 for RNA–protein interaction studies using the MS2-MCP system. HEK293T cells were transfected with FS2-tagged MS2 circular RNA and/or FLAG-tagged MCP protein. FS2-mediated pulldown successfully enriched MCP, as confirmed by Western blot analysis, demonstrating the feasibility of FS2 in capturing structured RNA-protein complexes. (**C**) Schematic illustration of the FS2-tagged DRACH construct, in which FS2 was incorporated at the 5′ end of six tandem DRACH motifs and expressed under the CMV promoter. (**D**) FS2-tagged DRACH enables the selective pulldown of endogenous YTHDF2 from m^6^A-modified RNA. Wild-type and *METTL3* knockout mESCs were transfected with FS2-tagged DRACH circular constructs, and FS2-mediated purification selectively enriched YTHDF2 in an m^6^A-dependent manner. Western blot analysis confirmed YTHDF2 enrichment in wild-type cells but not in *METTL3* knockout cells, validating the specificity of the FS2-tagged DRACH system for studying m^6^A–protein interactions. The schematic illustration in panel A was created in BioRender. Jaffrey, S. R. (2026) https://BioRender.com/shc5al4, and the schematic illustration in panel C was created in BioRender. Jaffrey, S. R. (2026) https://BioRender.com/789ojvk.

In many cases, the goal is to pull down an RNA from a cellular extract, along with its bound proteins. To test the ability of FS2 to pull down RNA–protein complexes, we first tested its performance in a well-characterized high-affinity interaction: the MS2-MCP system. The MS2 RNA hairpin binds the MS2 bacteriophage coat protein (MCP) with nanomolar affinity (*K*_d_ = 0.038 nM [20]), making it an ideal model for evaluating FS2’s efficiency in RNA-protein pulldowns (Fig. 5A).

For this experiment, we expressed a circular FS2-tagged MS2 RNA construct to maximize RNA expression levels. HEK293T cells (∼10^7^) were co-transfected with FS2-tagged MS2 RNA and FLAG-tagged MCP protein. After 48 h, cells were harvested and lysed, and the clarified extracts were subjected to FS2-based affinity purification under the same conditions optimized for FS2-IRES RNA (see Materials and methods). Binding was carried out at 4°C for 100 min, followed by three washes with binding buffer to minimize non-specific interactions. Bound material was eluted with EDTA under standard conditions.

Western blot analysis of the eluates confirmed strong enrichment of FLAG–MCP in FS2 pulldowns (Fig. 5B; Supplementary Fig. S9). Minimal background was observed in the negative controls, which included: (i) untransfected cells, (ii) cells transfected only with FS2-tagged MS2 RNA, and (iii) cells transfected only with FLAG–MCP. FLAG–MCP was detected in the eluate only from cells co-expressing both the protein and the circular FS2–MS2 RNA, indicating that efficient MCP recovery depends on the presence of FS2-tagged RNA. Protein gel staining of the eluates further supported enrichment of MCP in FS2–MS2 pulldowns (Supplementary Fig. S10).

Together, these results establish FS2 as an effective affinity tag for isolating RNA–protein complexes directly from mammalian cell extracts.

#### FS2-tagged m^6^A RNA pulldown of endogenous YTHDF2

As an additional example of pulldown of RNA–protein complexes, we tested RNA containing N6-methyladenosine (m^6^A), the most prevalent internal mRNA modification [21, 22]. m^6^A is deposited by the METTL3 complex at DRACH motifs (D = A/G/U, R = A/G, H = A/C/U) [23]. YTHDF2 is a key m^6^A-binding protein that recognizes these motifs and regulates RNA decay [24, 25]. Compared to the MS2–MCP interaction (*K*_d_ = 0.038 nM), YTHDF2 (*K*_d_ = 162 nM) exhibits an affinity ∼4 200-fold weaker with m^6^A RNA, making its purification potentially more challenging.

To test whether FS2 could pull down YTHDF2, despite its lower affinity compared to MS2, we expressed a FS2-tagged RNA containing the consensus motif for m^6^A formation. This motif, termed “DRACH” (D = A, G, U; R = A, G; H = A, C, U), was present in 6 copies (“6 × DRACH motif”) in 62 nt. An FS2 construct was incorporated at the 5′ end (Fig. 5C). We expressed a circular form of the FS2-tagged DRACH construct, which is expected to be expressed at much higher levels [10, 26]. The construct was expressed using a CMV Pol II promoter in wild-type and *METTL3* knockout mESCs to allow or prevent m^6^A deposition, respectively.

We next performed pulldown using the same conditions used for the MS2-MCP pulldown. Despite the relatively weak YTHDF2–m^6^A interaction, endogenous YTHDF2 was robustly enriched in the FS2 pulldown from lysates derived from wild-type mESCs (Fig. 5D). As a control, we found that no YTHDF2 pulldown was observed in the FS2 pulldown from lysates prepared from *METTL3* knockout mESCs. These results confirm that YTHDF2 pulldown depended strictly on m^6^A modification of DRACH motifs, not an artifact of nonspecific RNA–protein binding. Together with the MS2 results, these data establish FS2 as a versatile affinity tag that can support pulldown across an affinity spectrum, from strong interactions to physiologically relevant but weak RNA–protein associations.

## Discussion

RNA affinity purification is a crucial tool in molecular biology, yet existing methods remain limited by sequence dependence, protein immobilization requirements, and background contamination. In this study, we developed the FS2 tag, a high-affinity tag for an RNA purification system that incorporates a bivalent D8 aptamer within the F30 scaffold. Although D8 is known to bind Sephadex beads, its folding and function are impaired when inserted as an affinity tag into other RNA sequences. By using the F30 RNA folding scaffold, the D8 aptamer structure was stabilized, resulting in efficient Sephadex binding of D8-tagged RNAs. Furthermore, incorporating two D8 aptamers into the F30 scaffold enhanced the avidity of the complex, leading to faster binding kinetics, higher binding affinity, and improved overall RNA recovery. We show that this RNA affinity tag, termed FS2, enables efficient purification of RNA and RNA–protein complexes. Our findings demonstrate that FS2 provides a highly efficient tool for RNA pulldown that overcomes limitations with current approaches.

### FS2 improves RNA purification efficiency compared to existing methods

Current RNA affinity purification methods rely heavily on protein-based or hybridization-based strategies, both of which have fundamental drawbacks. The MS2 coat protein (MCP)-MS2 hairpin system requires recombinant protein immobilization, limiting binding capacity and increasing background contamination from endogenous RBPs. Similarly, streptavidin-based purification approaches often require additional biotinylation steps and may involve stringent wash conditions, potentially leading to RNA degradation. By contrast, FS2 enables protein-free RNA purification, eliminating the need for immobilized proteins and reducing non-specific interactions.

Compared to previously reported RNA-based affinity tags, such as streptavidin-binding aptamers [7], FS2 offers several advantages. Notably, FS2 directly binds to the Sephadex matrix without requiring an immobilized protein partner, thereby simplifying the purification workflow and reducing potential sources of background binding. Additionally, because Sephadex consists of a highly dense dextran network, FS2 may benefit from a large number of available binding sites, which may contribute to its binding capacity and purification efficiency. Sephadex is also relatively inexpensive, reusable, and compatible with denaturing buffers, which makes it broadly useful. Caution should be used when using other dextran-based supports since the D8 moiety selectively binds to glycosidic linkages found in certain Sephadex beads.

Other small molecule-binding aptamers have been developed for diverse applications, including RNA imaging, molecular sensing, and affinity-based targeting. For example, fluorogenic aptamers such as Spinach [27], Broccoli [28], and Mango [29] bind small-molecule fluorophores and have been widely used for RNA imaging. However, repurposing them as RNA purification tags would require access to suitable ligands and, for purification applications, immobilized ligand formats, which may limit cost-effectiveness, scalability, and broad practical use. In contrast, FS2 directly binds Sephadex, an inexpensive and widely available dextran-based matrix, without requiring an intermediate protein component or a specialized immobilized ligand. This direct matrix-binding property enables a simple, low-cost, and scalable protein-free RNA purification strategy.

Hybridization-based approaches, such as biotinylated antisense oligonucleotide pulldowns, require denaturing conditions, which may disrupt native RNA secondary structures and RNA–protein complexes. In contrast, FS2 purification is performed under physiological conditions, preserving RNA structure and interactions, making it particularly suitable for RNA–protein interaction studies and epitranscriptomics research.

The F30 scaffold plays a central role in FS2’s efficiency, forcing the D8 aptamer into a folded conformation, thus ensuring optimal presentation of its binding domain. F30 acts as an “insulator” that protects the D8 aptamer from nearby sequences that could base pair with portions of the D8 sequence, preventing D8 folding. Additionally, the bivalent D8 configuration within FS2 enhances binding affinity via avidity effects [30], significantly improving RNA binding to Sephadex. These structural improvements make FS2 a robust and efficient affinity tag for purifying structured and non-coding RNAs.

The ability to perform RNA purification in the presence of detergents is an important practical advantage, particularly for minimizing RNA degradation during extended handling. Sodium dodecyl sulfate (SDS) is commonly used to inhibit RNase activity by denaturing proteins, thereby protecting RNA integrity. However, such denaturing conditions can disrupt interactions of RNA with immobilized proteins, such as MCP. In contrast, FS2 retained robust binding to Sephadex even in the presence of SDS (Fig. 3E), indicating that RNA folding is unaffected by SDS and the FS2–Sephadex interaction remains highly stable even in the presence of SDS. This feature may be particularly beneficial in workflows requiring prolonged incubation or enhanced protection against RNase activity. Importantly, FS2 also remained functional in the presence of RIPA buffer (Supplementary Fig. S4), further supporting its compatibility with detergent-containing lysis conditions and broader applicability to mammalian cell lysate purification workflows.

### Elution strategies for FS2

A critical aspect of RNA-pulldown experiments is elution of the bound RNA. In some cases, only the bound proteins are desired, in which the protein can be directly eluted with SDS, or in the case of proteomic analyses, with proteases. However, if the RNA is desired with its bound proteins, milder elution conditions are preferable. One potential strategy is competitive elution using a dextran-containing buffer, which could compete with the Sephadex matrix and promote release of FS2-tagged RNA. In this study, we used a simpler approach based on magnesium chelation. We found that elution with 10 mM EDTA at 50°C for 10 min efficiently released FS2-tagged RNA and bound proteins from Sephadex beads. When recovery of RNA alone is desired, SDS is useful because it helps inhibit RNase activity and preserve RNA integrity. Dextran beads can potentially be reused after washing with guanidinium HCl or urea to remove bound RNA and protein.

### FS2 enables efficient RNA–protein complex purification

Beyond RNA isolation, FS2 facilitates the purification of RNA–protein complexes, expanding its utility in molecular biology research. Our results demonstrate that FS2-tagged RNAs containing DRACH motifs efficiently capture YTHDF2, a key reader of m^6^A-modified RNA, in an m^6^A-dependent manner. Unlike conventional RNA pulldown techniques, FS2 preserves native RNA modifications, making it an ideal tool for epitranscriptomics studies. The ability to selectively enrich m^6^A-modified transcripts and their associated proteins suggests that FS2 could be adapted to investigate other RNA modifications and RNA-binding proteins (RBPs). Because FS2 enables affinity purification of tagged RNAs directly from mammalian cells under native conditions, the platform may also be adaptable to transcriptome-wide RNA and proteome-wide interactome analyses using RNA sequencing and mass spectrometry-based approaches. In the current study, we focused on proof-of-concept validation of known RNA–protein interactions by Western blotting. Future studies will be needed to comprehensively evaluate specificity and background interactions in unbiased interactome profiling applications.

Another important direction will be to explore FS2’s lower affinity threshold, determining whether it can be applied to weaker or transient RNA–protein interactions.

### Dependence on Sephadex beads

FS2 purification relies on Sephadex beads, which provide strong and specific binding but are not widely used in high-throughput purification workflows. Sephadex beads are appealing due to the low cost and reusability of the beads. The development of magnetic beads containing dextran with the appropriate chain linkage could be useful for certain automated applications.

### Compatibility with longer, highly structured, and circular RNAs

Our results demonstrate that FS2 enables efficient purification of diverse RNA classes, including structured RNAs, long mRNA constructs, and circular RNAs. The successful optimization of FS2-tagged IRES RNA purification confirms that FS2 remains functional even in the presence of highly structured regions, such as internal ribosome entry sites (IRES), which adopt complex tertiary conformations essential for translation initiation. The ability of FS2 to efficiently recover IRES-containing RNAs highlights its robust binding capacity in structured RNA contexts.

Additionally, FS2 was successfully applied to both linear and circular RNA constructs, as demonstrated by FS2-tagged *MYC* purification using a circular RNA format. Circular RNAs, which lack free 5′ and 3′ ends, present unique structural challenges for purification, yet FS2-mediated enrichment remained highly efficient. This result suggests that FS2 is not dependent on RNA termini for binding accessibility, making it a promising tool for circular RNA studies.

Together, these results establish FS2 as a versatile RNA affinity tag suitable for structured RNAs, circular RNAs, and RNA–protein interaction studies. Future work should explore its application to full-length coding transcripts, long non-coding RNAs (lncRNAs), and other structured non-coding RNAs, as well as further optimization of FS2 positioning within different RNA architectures.

### Broader applications of FS2 in RNA biology

Beyond RNA purification, FS2 may also have potential applications in RNA epitranscriptomics, transcriptomics, and *in vivo* RNA tracking. Future studies may further expand FS2-based approaches for transcriptome-wide and proteome-wide interactome analyses. Additionally, FS2-based purification strategies could be applied to study RNA modifications beyond m^6^A, such as pseudouridylation or 5-methylcytosine, broadening its impact in RNA biology.

While FS2 represents a major advancement in RNA purification, further optimizations could enhance its scalability and adaptability. Future studies could also assess the functional integrity of FS2-purified RNAs in reverse transcription, translation, splicing, and intracellular RNA processing, confirming that FS2 does not interfere with essential RNA functions.

## Conclusion

In summary, FS2 represents a highly efficient, protein-free RNA affinity tag that overcomes the limitations of existing purification methods. By integrating a bivalent D8 aptamer within the F30 scaffold, FS2 achieves enhanced RNA stability, improved binding efficiency, and broad applicability in RNA purification and RNA–protein interaction studies. Our findings establish FS2 as a versatile tool for RNA biochemistry, epitranscriptomics, and transcriptomics research, with potential applications in high-throughput RNA sequencing and modification profiling.

## Supplementary Material

gkag735_Supplemental_File

## Data Availability

The data underlying this article are available in the article and in its online supplementary data.
