# Supplementary material for: A multivalent RNA affinity tag enables selective purification of tagged RNAs and bound proteins
Source: Nucleic Acids Res. 2026 Jul 30;54(14):gkag735. doi: 10.1093/nar/gkag735 (PMC13421773; doi:10.1093/nar/gkag735)
Supplement: gkag735_Supplemental_File [file gkag735_supplemental_file.pdf]

Supplementary Information

A multivalent RNA affinity tag enables selective purification of tagged RNAs and bound proteins

Ki Sung Park, Sourav Kumar Dey, Rohit Nalavade, Aashiq H. Mirza, Qian Hou, Mateo A. Martinez Roque, Maxim Oleynikov, Jacob L. Litke, and Samie R. Jaffrey*

Department of Pharmacology, Weill Cornell Medicine, Cornell University, New York, NY 10065, USA

* To whom correspondence should be addressed. Email: srj2003@med.cornell.edu

Contents

Table S1. Nucleotide sequences used in this study.

Table S2. Amino acid and nucleotide sequences of FLAG-MCP constructs used in this study.

Figure S1. Quantification of D8- and FS1-tagged RNA binding across multiple RNA contexts.

Figure S2. Untagged IRES RNA does not show enrichment in the elution fraction after prolonged incubation.

Figure S3. mFold secondary structure prediction of FS4.

Figure S4. Efficient FS2-mediated RNA purification under RIPA-containing conditions.

Figure S5. Comparison of circular and linear MYC RNAs following FS2-tag affinity purification.

Figure S6. Comparison of circular D8- and FS2-tagged MYC RNA purification.

Figure S7. FS2 enables purification of a long 1.6 kb mRNA reporter in mammalian cells.

Figure S8. FS4-mediated enrichment of low-abundance reporter mRNA from cellular RNA.

Figure S9. Uncropped Western blot images corresponding to Figure 5B and 5D.

Figure S10. Protein gel staining of eluates from FS2–MS2 pulldown.

Table S1. Nucleotide sequences used in this study

Name	Sequence (5′ to 3′)
**D8 aptamer**	TCCGAGTAATTTACGTTTTGATACGGTTGCGGA
**FS1**	GGAGCCATGTGTATGTGGTCCGAGTAATTTACGTTTTGATACGGTTGCG GACCACATACTCTGATGATCCTTCGGGATCATTCATGGCTCC
**FS2**	GGAGCCATGTGTATGTGGTCCGAGTAATTTACGTTTTGATACGGTTGCG GACCACATACTCTGATGATCCTCCGAGTAATTTACGTTTTGATACGGTTG CGGAGGATCATTCATGGCTCC
**FS2-tagged Gaussia Luciferase**	GCCACCATGGGAGTCAAAGTTCTGTTTGCCCTGATCTGCATCGCTGTGG CCGAGGCCAAGCCCACCGAGAACAACGAAGACTTCAACATCGTGGCCG TGGCCAGCAACTTCGCGACCACGGATCTCGATGCTGACCGCGGGAAGT TGCCCGGCAAGAAGCTGCCGCTGGAGGTGCTCAAAGAGTTGGAAGCCA ATGCCCGGAAAGCTGGCTGCACCAGGGGCTGTCTGATCTGCCTGTCCC ACATCAAGTGCACGCCCAAGATGAAGAAGTTCATCCCAGGACGCTGCCA CACCTACGAAGGCGACAAAGAGTCCGCACAGGGCGGCATAGGCGAGG CGATCGTCGACATTCCTGAGATTCCTGGGTTCAAGGACTTGGAGCCCTT GGAGCAGTTCATCGCACAGGTCGATCTGTGTGTGGACTGCACAACTGG CTGCCTCAAAGGGCTTGCCAACGTGCAGTGTTCTGACCTGCTCAAGAAG TGGCTGCCGCAACGCTGTGCGACCTTTGCCAGCAAGATCCAGGGCCAG GTGGACAAGATCAAGGGGGCCGGTGGTGACTAAGCGGCCGCAAAAGGA GCCATGTGTATGTGGTCCGAGTAATTTACGTTTTGATACGGTTGCGGAC CACATACTCTGATGATCCTCCGAGTAATTTACGTTTTGATACGGTTGCGG AGGATCATTCATGGCTCC
**FS2-tagged NanoLuc**	ATGGTCTTCACACTCGAAGATTTCGTTGGGGACTGGCGACAGACAGCCG GCTACAACCTGGACCAAGTCCTTGAACAGGGAGGTGTGTCCAGTTTGTT TCAGAATCTCGGGGTGTCCGTAACTCCGATCCAAAGGATTGTCCTGAGC GGTGAAAATGGGCTGAAGATCGACATCCATGTCATCATCCCGTATGAAG GTCTGAGCGGCGACCAAATGGGCCAGATCGAAAAAATTTTTAAGGTGGT GTACCCTGTGGATGATCATCACTTTAAGGTGATCCTGCACTATGGCACA CTGGTAATCGACGGGGTTACGCCGAACATGATCGACTATTTCGGACGGC CGTATGAAGGCATCGCCGTGTTCGACGGCAAAAAGATCACTGTAACAGG GACCCTGTGGAACGGCAACAAAATTATCGACGAGCGCCTGATCAACCCC GACGGCTCCCTGCTGTTCCGAGTAACCATCAACGGAGTGACCGGCTGG CGGCTGTGCGAACGCATTCTGGCGTAATTCTAGAGTCGGGGCGGCGGA GCCATGTGTATGTGGTCCGAGTAATTTACGTTTTGATACGGTTGCGGAC CACATACTCTGATGATCCTCCGAGTAATTTACGTTTTGATACGGTTGCGG AGGATCATTCATGGCTCC

**FS2-tagged mCherry**	ATGGTGAGCAAGGGCGAGGAGGATAACATGGCCATCATCAAGGAGTTC<br>ATGCGCTTCAAGGTGCACATGGAGGGCTCCGTGAACGGCCACGAGTTC<br>GAGATCGAGGGCGAGGGCGAGGGCCGCCCCTACGAGGGCACCCAGAC<br>CGCCAAGCTGAAGGTGACCAAGGGTGGCCCCCTGCCCTTCGCCTGGGA<br>CATCCTGTCCCCTCAGTTCATGTACGGCTCCAAGGCCTACGTGAAGCAC<br>CCCGCCGACATCCCCGACTACTTGAAGCTGTCCTTCCCCGAGGGCTTCA<br>AGTGGGAGCGCGTGATGAACTTCGAGGACGGCGGCGTGGTGACCGTG<br>ACCCAGGACTCCTCCCTGCAGGACGGCGAGTTCATCTACAAGGTGAAG<br>CTGCGCGGCACCAACTTCCCCTCCGACGGCCCCGTAATGCAGAAGAAG<br>ACCATGGGCTGGGAGGCCTCCTCCGAGCGGATGTACCCCGAGGACGG<br>CGCCCTGAAGGGCGAGATCAAGCAGAGGCTGAAGCTGAAGGACGGCG<br>GCCACTACGACGCTGAGGTCAAGACCACCTACAAGGCCAAGAAGCCCG<br>TGCAGCTGCCCGGCGCCTACAACGTCAACATCAAGTTGGACATCACCTC<br>CCACAACGAGGACTACACCATCGTGGAACAGTACGAACGCGCCGAGGG<br>CCGCCACTCCACCGGCGGCATGGACGAGCTGTACAAGTAACTCGAGAA<br>TACTGCGGCCGCGTTACAAGGAGCCATGTGTATGTGGTCCGAGTAATTT<br>ACGTTTTGATACGGTTGCGGACCACATACTCTGATGATCCTCCGAGTAAT<br>TTACGTTTTGATACGGTTGCGGAGGATCATTCATGGCTCC
**FS2-tagged IRES**	GCGAATTAATTCCGGTTATTTTCCACCATATTGCCGTCTTTTGGCAATGT<br>GAGGGCCCGGAAACCTGGCCCTGTCTTCTTGACGAGCATTCCTAGGGG<br>TCTTTCCCCTCTCGCCAAAGGAATGCAAGGTCTGTTGAATGTCGTGAAG<br>GAAGCAGTTCCTCTGGAAGCTTCTTGAAGACAAACAACGTCTGTAGCGA<br>CCCTTTGCAGGCAGCGGAACCCCCCACCTGGCGACAGGTGCCTCTGCG<br>GCCAAAAGCCACGTGTATAAGATACACCTGCAAAGGCGGCACAACCCCA<br>GTGCCACGTTGTGAGTTGGATAGTTGTGGAAAGAGTCAAATGGCTCACC<br>TCAAGCGTATTCAACAAGGGGCTGAAGGATGCCCAGAAGGTACCCCATT<br>GTATGGGATCTGATCTGGGGCCTCGGTGCACATGCTTTACATGTGTTTA<br>GTCGAGGTTAAAAAACGTCTAGGCCCCCCGAACCACGGGGACGTGGTT<br>TTCCTTTGAAAAACACGATGATAATATGGCCACCACCCATATGGGATCCG<br>AATTCGATATCTTAATTAAGCTGCAGGAGCTCGTCGACGCGGCCGCACT<br>CGAGCACCACCACCACCACCACTGAGATCTGGAGCCATGTGTATGTGGT<br>CCGAGTAATTTACGTTTTGATACGGTTGCGGACCACATACTCTGATGATC<br>CTCCGAGTAATTTACGTTTTGATACGGTTGCGGAGGATCATTCATGGCTC<br>C
**FS2-tagged *MYC* mRNA fragment (U6 promoter)**	GAGGGCCTATTTCCCATGATTCCTTCATATTTGCATATACGATACAAGGC<br>TGTTAGAGAGATAATTAGAATTAATTTGACTGTAAACACAAAGATATTAGT<br>ACAAAATACGTGACGTAGAAAGTAATAATTTCTTGGGTAGTTTGCAGTTT<br>TAAAATTATGTTTTAAAATGGACTATCATATGCTTACCGTAACTTGAAAGT<br>ATTTCGATTTCTTGGCTTTATATATCTTGTGGAAAGGACGAAACACCGTG<br>CTCGCTTCGGCAGCACATATACTAGTCGACGGAGCCATGTGTATGTGGT<br>CCGAGTAATTTACGTTTTGATACGGTTGCGGACCACATACTCTGATGATC<br>CTCCGAGTAATTTACGTTTTGATACGGTTGCGGAGGATCATTCATGGCTC<br>CCACAATGAATCAAAAACATCATCATCCAGGACTGTATGTGGAGCGGCT<br>TCTCGGCCGCCTCTAGAGCGCACTTCGGTGCGCTTTT

**Circular FS2-tagged *MYC* mRNA fragment (U6 promoter)**	GAGGGCCTATTTCCCATGATTCCTTCATATTTGCATATACGATACAAGGC<br>TGTTAGAGAGATAATTAGAATTAATTTGACTGTAAACACAAAGATATTAGT<br>ACAAAATACGTGACGTAGAAAGTAATAATTTCTTGGGTAGTTTGCAGTTT<br>TAAAATTATGTTTTAAAATGGACTATCATATGCTTACCGTAACTTGAAAGT<br>ATTTCGATTTCTTGGCTTTATATATCTTGTGGAAAGGACGAAACACCGTG<br>CTCGCTTCGGCAGCACATATACTAGTCGACGGGCCGCACTCGCCGGTC<br>CCAAGCCCGGATAAAATGGGAGGGGGCGGGAAACCGCCTAACCATGCC<br>GAGTGCGGCCGCGGAGCCATGTGTATGTGGTCCGAGTAATTTACGTTTT<br>GATACGGTTGCGGACCACATACTCTGATGATCCTCCGAGTAATTTACGTT<br>TTGATACGGTTGCGGAGGATCATTCATGGCTCCCACAATGAATCAAAAA<br>CATCATCATCCAGGACTGTATGTGGAGCGGCTTCTCGGCCGCCGTGGC<br>CGCGGTCGGCGTGGACTGTAGAACACTGCCAATGCCGGTCCCAAGCCC<br>GGATAAAAGTGGAGGGTACAGTCCACGCTCTAGAGCGCACTTCGGTGC<br>GCTTTT
**Circular FS2-tagged MS2 (U6 promoter)**	GAGGGCCTATTTCCCATGATTCCTTCATATTTGCATATACGATACAAGGC<br>TGTTAGAGAGATAATTAGAATTAATTTGACTGTAAACACAAAGATATTAGT<br>ACAAAATACGTGACGTAGAAAGTAATAATTTCTTGGGTAGTTTGCAGTTT<br>TAAAATTATGTTTTAAAATGGACTATCATATGCTTACCGTAACTTGAAAGT<br>ATTTCGATTTCTTGGCTTTATATATCTTGTGGAAAGGACGAAACACCGTG<br>CTCGCTTCGGCAGCACATATACTAGTCGACGGGCCGCACTCGCCGGTC<br>CCAAGCCCGGATAAAATGGGAGGGGGCGGGAAACCGCCTAACCATGCC<br>GAGTGCGGCCGCGGAGCCATGTGTATGTGGTCCGAGTAATTTACGTTTT<br>GATACGGTTGCGGACCACATACTCTGATGATCCTCCGAGTAATTTACGTT<br>TTGATACGGTTGCGGAGGATCATTCATGGCTCCCACAATGAGCACATGA<br>GGATCACCCATGTGCGTGGCCGCGGTCGGCGTGGACTGTAGAACACTG<br>CCAATGCCGGTCCCAAGCCCGGATAAAAGTGGAGGGTACAGTCCACGC<br>TCTAGAGCGCACTTCGGTGCGCTTTT
**Linear FS2-tagged DRACH (CMV promoter)**	CGTTACATAACTTACGGTAAATGGCCCGCCTGGCTGACCGCCCAACGAC<br>CCCCGCCCATTGACGTCAATAATGACGTATGTTCCCATAGTAACGCCAAT<br>AGGGACTTTCCATTGACGTCAATGGGTGGAGTATTTACGGTAAACTGCC<br>CACTTGGCAGTACATCAAGTGTATCATATGCCAAGTACGCCCCCTATTGA<br>CGTCAATGACGGTAAATGGCCCGCCTGGCATTATGCCCAGTACATGACC<br>TTATGGGACTTTCCTACTTGGCAGTACATCTACGTATTAGTCATCGCTAT<br>TACCATGGTGATGCGGTTTTGGCAGTACATCAATGGGCGTGGATAGCGG<br>TTTGACTCACGGGGATTTCCAAGTCTCCACCCCATTGACGTCAATGGGA<br>GTTTGTTTTGGCACCAAAATCAACGGGACTTTCCAAAATGTCGTAACAAC<br>TCCGCCCCATTGACGCAAATGGGCGGTAGGCGTGTACGGTGGGAGGTC<br>TATATAAGCAGAGCTGGTTTAGTGAACCGTCAGATCCGCTAGCGGAGCC<br>ATGTGTATGTGGTCCGAGTAATTTACGTTTTGATACGGTTGCGGACCACA<br>TACTCTGATGATCCTCCGAGTAATTTACGTTTTGATACGGTTGCGGAGGA<br>TCATTCATGGCTCCCACAATGAATCAAAGGACTCATCAGGACTGACGGA<br>CTGTATGGACTTTCTGGACTGCCGGACTCCGATCG
**Circular FS2-tagged DRACH (CMV promoter)**	CGTTACATAACTTACGGTAAATGGCCCGCCTGGCTGACCGCCCAACGAC<br>CCCCGCCCATTGACGTCAATAATGACGTATGTTCCCATAGTAACGCCAAT<br>AGGGACTTTCCATTGACGTCAATGGGTGGAGTATTTACGGTAAACTGCC<br>CACTTGGCAGTACATCAAGTGTATCATATGCCAAGTACGCCCCCTATTGA

	CGTCAATGACGGTAAATGGCCCGCCTGGCATTATGCCCAGTACATGACC<br>TTATGGGACTTTCCTACTTGGCAGTACATCTACGTATTAGTCATCGCTAT<br>TACCATGGTGATGCGGTTTTGGCAGTACATCAATGGGCGTGGATAGCGG<br>TTTGACTCACGGGGATTTCCAAGTCTCCACCCCATTGACGTCAATGGGA<br>GTTTGTTTTGGCACCAAAATCAACGGGACTTTCCAAAATGTCGTAACAAC<br>TCCGCCCCATTGACGCAAATGGGCGGTAGGCGTGTACGGTGGGAGGTC<br>TATATAAGCAGAGCTGCTTCGGCAGCACATATACTAGTCGACGGGCCGC<br>ACTCGCCGGTCCCAAGCCCGGATAAAATGGGAGGGGGCGGGAAACCG<br>CCTAACCATGCCGAGTGCGGCCGCGGAGCCATGTGTATGTGGTCCGAG<br>TAATTTACGTTTTGATACGGTTGCGGACCACATACTCTGATGATCCTCCG<br>AGTAATTTACGTTTTGATACGGTTGCGGAGGATCATTCATGGCTCCCACA<br>ATGAATCAAAGGACTCATCAGGACTGACGGACTGTATGGACTTTCTGGA<br>CTGCCGGACTCCGAGTGGCCGCGGTCGGCGTGGACTGTAGAACACTGC<br>CAATGCCGGTCCCAAGCCCGGATAAAAGTGGAGGGTACAGTCCACGCT<br>CTAGAGCGCACTTCGGTGCGCTTTT

* Colors indicate the position of the F30 scaffold: Red, 5′ F30; Green, Mid F30; Purple, 3′ F30.

Table S2. Amino acid and nucleotide sequences of FLAG-MCP constructs used in this study

Name	Sequence
**3×FLAG–MCP (amino acid)**	MDYKDHDGDYKDHDIDYKDDDDKASNFTQFVLVDNGGTGDVTVAPSNFA<br>NGVAEWISSNSRSQAYKVTCSVRQSSAQNRKYTIKVEVPKVATQTVGGV<br>ELPVAAWRSYLNMELTIPIFATNSDCELIVKAMQGLLKDGNPIPSAIAANSGI<br>Y*
**3×FLAG–MCP (coding sequence, 5′→3′)**	ATGGATTATAAAGACCATGACGGGGATTACAAGGACCATGACATAGAC<br>TACAAAGACGATGATGATAAGGCATCAAACTTCACTCAGTTTGTCCTGG<br>TGGACAACGGAGGCACTGGGGACGTAACGGTTGCTCCCAGCAACTTC<br>GCGAATGGCGTTGCTGAGTGGATTTCTTCCAATAGTCGCTCCCAGGCC<br>TATAAGGTAACTTGTTCCGTGCGACAAAGCTCAGCGCAAAACAGGAAA<br>TATACTATAAAGGTTGAAGTACCGAAAGTAGCGACGCAAACGGTTGGG<br>GGGGTTGAGCTGCCCGTTGCTGCATGGAGGTCTTACTTGAATATGGAA<br>CTTACCATACCCATTTTTGCTACGAACAGCGACTGCGAGCTGATCGTG<br>AAAGCCATGCAGGGGCTCTTGAAGGACGGCAATCCTATCCCATCTGCG<br>ATTGCTGCGAACAGTGGGATCTATTAA

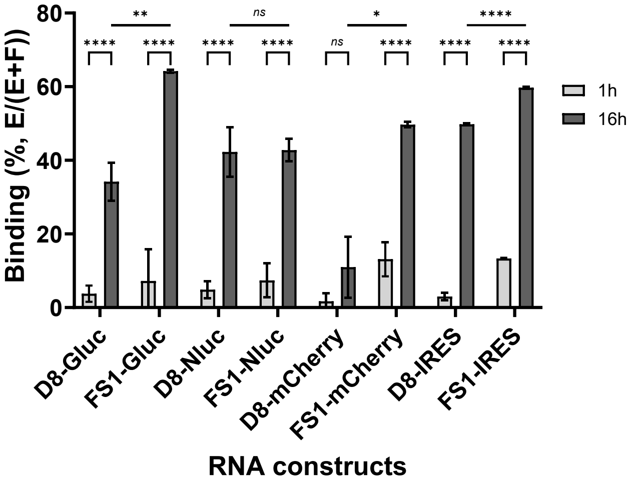

Figure S1. Quantification of D8- and FS1-tagged RNA binding across multiple RNA contexts. Binding efficiencies of D8- and FS1-tagged Nluc, Gluc, mCherry, and IRES RNAs were quantified following 1 h or 16 h incubation with Sephadex beads. Binding efficiency was calculated as the band intensity in the elution fraction divided by the combined band intensities in the flow-through and elution fractions, E/(F+E), using densitometric analysis in ImageJ. Across all RNA contexts tested, prolonged incubation substantially increased the fraction of D8- and FS1-tagged RNAs detected in the elution fraction. Data are shown as mean ± SD from three independent experiments. Statistical significance for 1 h versus 16 h incubation conditions was determined by two-way ANOVA followed by Šídák's multiple comparisons test. Statistical significance between D8 and FS1 constructs at 16 h incubation was determined for each RNA context using unpaired two-tailed t-tests. ns, not significant; *P < 0.05, **P < 0.01, ****P < 0.0001.

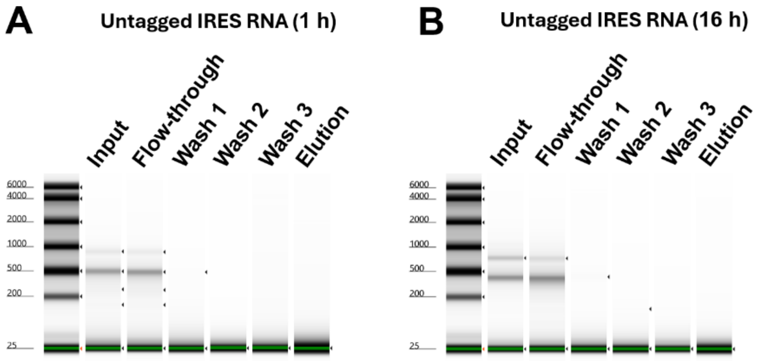

Figure S2. Untagged IRES RNA does not show enrichment in the elution fraction after prolonged incubation. Untagged IRES RNA was incubated with Sephadex beads under the same conditions used for D8- and FS1-tagged RNAs (1 h and 16 h at 4°C). RNA was analyzed in the input, flow-through, wash, and elution fractions using a microfluidic capillary electrophoresis system (Agilent TapeStation). While RNA was readily detected in the input and flow-through fractions, no appreciable enrichment was observed in the elution fraction, indicating that RNA binding to Sephadex is dependent on the presence of the D8 or FS1 tag.

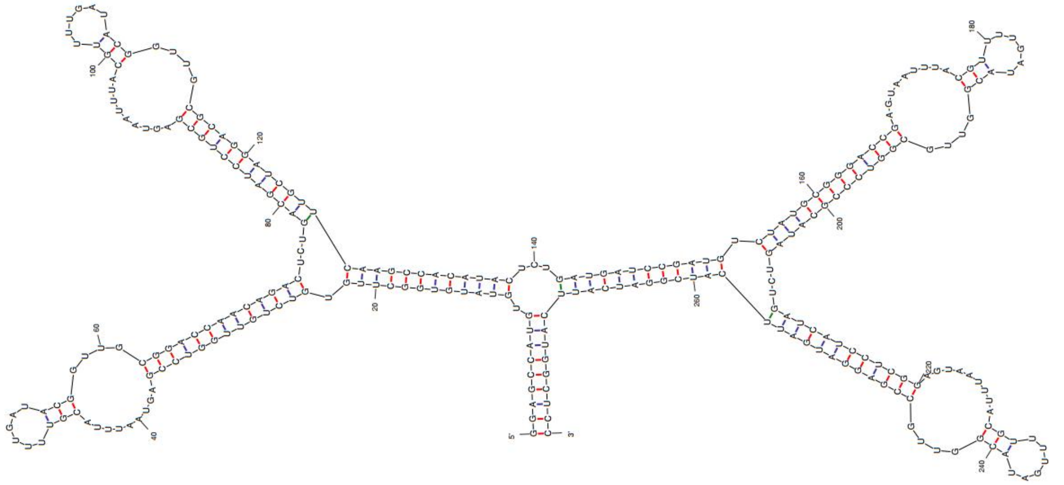

Figure S3. mFold secondary structure prediction of FS4.

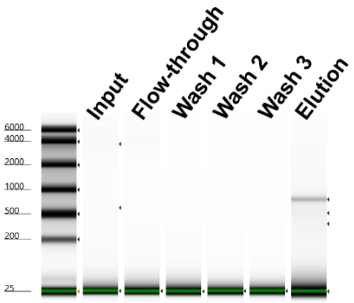

Figure S4. Efficient FS2-mediated RNA purification under RIPA-containing conditions. Binding buffer was supplemented with 1× RIPA buffer, and the $MgCl_2$ concentration was increased to 10 mM to compensate for EDTA present in RIPA. FS2-tagged IRES RNA was efficiently recovered in the elution fraction, demonstrating that FS2 remains functional under detergent-containing conditions. The reduced signal observed in the input and wash fractions is likely due to detergent effects on RNA detection during TapeStation analysis, rather than reduced RNA input or impaired FS2 binding, as described in Methods.

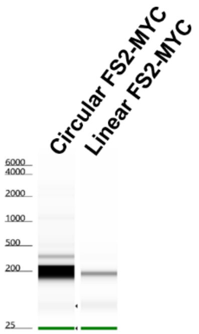

Figure S5. Comparison of circular and linear *MYC* RNAs following FS2-tag affinity purification. Bioanalyzer electropherogram images showing purified FS2-tagged circular *MYC* RNA and linear *MYC* RNA after two rounds of affinity purification, alongside an RNA ladder with sizes indicated in nucleotides. The circular *MYC* RNA exhibited a higher signal compared to the linear counterpart, as evidenced by a more intense band at the expected size. This difference likely reflects increased expression and/or stability of circular RNA compared to the linear form. These results demonstrate that FS2-mediated purification is compatible with both linear and circular RNA constructs.

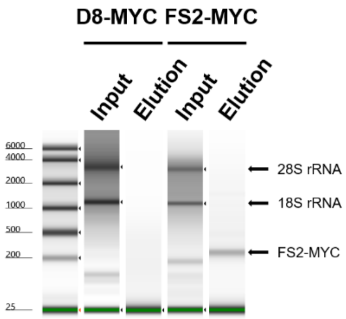

Figure S6. Comparison of circular D8- and FS2-tagged *MYC* RNA purification. Circular *MYC* RNA constructs containing either the original D8 aptamer or the FS2 tag were subjected to Sephadex-based affinity purification under identical conditions. Input and elution fractions were analyzed using microfluidic capillary electrophoresis. FS2-tagged circular *MYC* RNA was readily detected in the elution fraction, whereas D8-tagged *MYC* RNA showed little to no detectable recovery, indicating that FS2 substantially improves purification efficiency in this cellular circular RNA context.

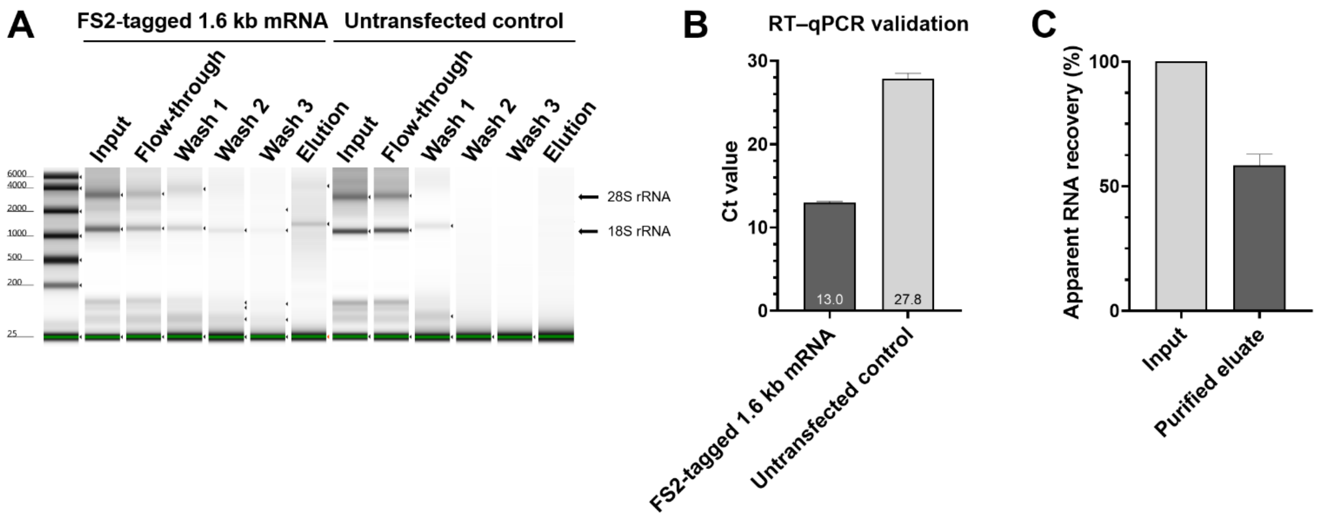

Figure S7. FS2 enables purification of a long 1.6 kb mRNA reporter in mammalian cells. (A) Cells were transfected with an FS2-tagged 1.6 kb mRNA construct, and RNA was purified using Sephadex beads. An RNA band corresponding to the expected size of the reporter mRNA was observed in the elution fraction from transfected cells, whereas no detectable signal was observed in untransfected controls. (B) RT–qPCR analysis of purified eluates from FS2-tagged 1.6 kb mRNA-expressing cells and untransfected controls. Lower Ct values indicate higher abundance of purified target RNA. Bars represent mean Ct ± SD from triplicate qPCR measurements. (C) RT–qPCR-based estimation of apparent recovery following FS2-mediated purification of the 1.6 kb reporter mRNA. RT–qPCR analysis estimated an apparent recovery of 58.4 ± 4.4%, indicating efficient recovery of the long mRNA target. Bars represent mean ± SD from triplicate qPCR measurements.

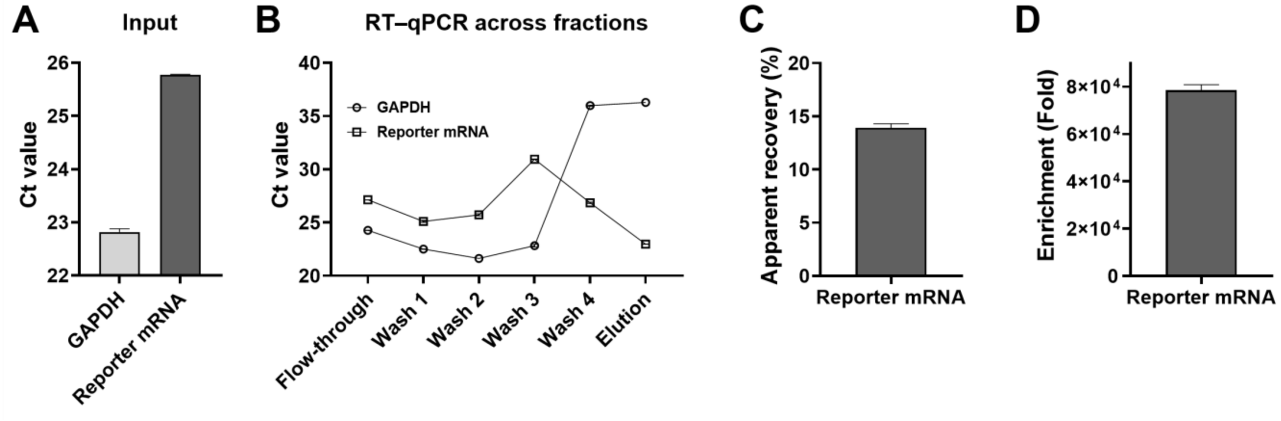

Figure S8. FS4-mediated enrichment of low-abundance reporter mRNA from cellular RNA. (A) RT–qPCR analysis comparing the abundance of FS4 reporter mRNA and *GAPDH* in the input sample. Ct values are shown for each target. (B) RT–qPCR analysis of *GAPDH* and FS4 reporter mRNA across purification fractions. The FS4 reporter mRNA showed the lowest Ct value in the elution fraction, consistent with recovery of the tagged reporter mRNA, whereas *GAPDH* showed high Ct values in the elution fraction, indicating minimal nonspecific carryover. (C) Apparent recovery of FS4 reporter mRNA in the first elution fraction after correction for total input and elution volumes. (D) Fold enrichment of FS4 reporter mRNA over *GAPDH* in the first elution fraction, calculated by the ΔΔCt method.

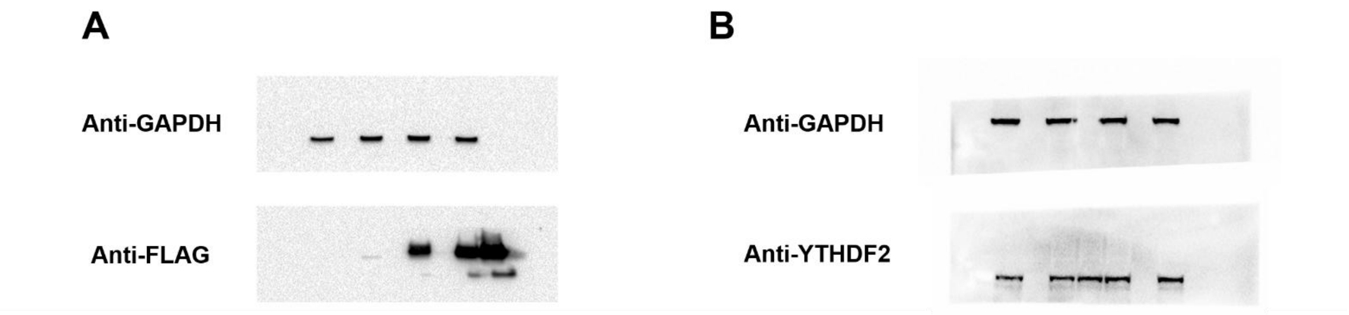

Figure S9. Uncropped Western blot images corresponding to Figure 5B and 5D. (A) Uncropped blot images corresponding to the MS2-MCP pulldown experiment shown in Figure 5B. (B) Uncropped blot images corresponding to the m^{6}A-dependent YTHDF2 pulldown experiment shown in Figure 5D. All bands shown in the main figures were derived from these uncropped blots.

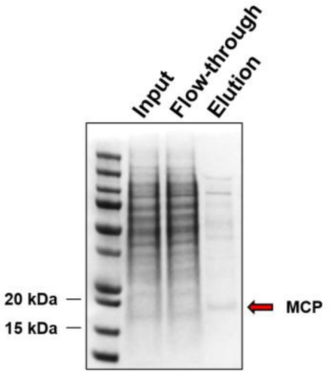

Figure S10. Protein gel staining of eluates from FS2–MS2 pulldown. HEK293T cells were co-transfected with circular FS2–MS2 RNA and FLAG–MCP, and eluates were resolved by SDS–PAGE followed by Coomassie staining. A prominent band corresponding to FLAG–MCP (red arrow) was enriched in the FS2 pulldown sample.
